# Adaptive constrained constructive optimisation for complex vascularisation processes

**DOI:** 10.1038/s41598-021-85434-9

**Published:** 2021-03-17

**Authors:** Gonzalo Daniel Maso Talou, Soroush Safaei, Peter John Hunter, Pablo Javier Blanco

**Affiliations:** 1 Department of Mathematical and Computational Methods, National Laboratory for Scientific Computing, Petrópolis, Brazil; 2 National Institute of Science and Technology in Medicine Assisted by Scientific Computing (INCT-MACC), São Paulo, Brazil; 3 Auckland Bioengineering Institute, The University of Auckland, Auckland, New Zealand

**Keywords:** Computational models, Computer modelling, Angiogenesis

## Abstract

Mimicking angiogenetic processes in vascular territories acquires importance in the analysis of the multi-scale circulatory cascade and the coupling between blood flow and cell function. The present work extends, in several aspects, the Constrained Constructive Optimisation (CCO) algorithm to tackle complex automatic vascularisation tasks. The main extensions are based on the integration of adaptive optimisation criteria and multi-staged space-filling strategies which enhance the modelling capabilities of CCO for specific vascular architectures. Moreover, this vascular outgrowth can be performed either from scratch or from an existing network of vessels. Hence, the vascular territory is defined as a partition of vascular, avascular and carriage domains (the last one contains vessels but not terminals) allowing one to model complex vascular domains. In turn, the multi-staged space-filling approach allows one to delineate a sequence of biologically-inspired stages during the vascularisation process by exploiting different constraints, optimisation strategies and domain partitions stage by stage, improving the consistency with the architectural hierarchy observed in anatomical structures. With these features, the aDaptive CCO (DCCO) algorithm proposed here aims at improving the modelled network anatomy. The capabilities of the DCCO algorithm are assessed with a number of anatomically realistic scenarios.

## Introduction

In the field of cardiovascular research, many circulatory diseases found in global or regional circulations begin with a dysfunctional chemo-mechanical pairing that governs the interaction between peripheral (arteriolar and capillary) blood flow and cellular functions^1–9^. Manifestation of abnormal signals in mid- and large-sized vessels and in whole-organ function is a consequence of the two-way coupling between growth and remodelling, and even loss of functional response of arterioles and capillaries, and the global systemic hemodynamic environment^10–15^. This interplay between peripheral and central vascular mechanics, mediated by proper chemical balances at the cell level and by blood pressure, blood flow and wall shear stress at the systemic level, is a clear example of the multi-scale nature of the circulation.

Because of the highly variable and diverse cell expression, the study of such multi-scale problems requires a proper mapping between cell function and cell location with the architecture of vascular territories^16–20^. Experimental approaches to answering questions involving such intricate mechanisms have a limited range of spatial scales. Therefore, mathematical models and computational simulations of the circulation and its coupling with cellular function provide powerful tools to push the boundaries of cardiovascular research^21–26^. However, the realisation of the domain of definition at a microvascular level is required for the blood flow models and cell coupling. This can be achieved by resorting to imaging techniques, which are capable of delivering high resolution images of small vessels in animal models. However, even with the power of these tools, their applicability to different vascular territories is rather constrained, as is the translation to humans^27–30^.

A solution to circumvent the lack of information about the vascular architecture of peripheral beds was found through the development of methodologies aimed at generating vascular networks in an automatic manner, driven by physiological criteria and in compliance with a set of morphometric constraints and empirical laws. The so-generated networks of vessels can be statistically consistent with the anatomical descriptions in the sense that they can reproduce the main topological features and functional response observed in vascular networks.

There are two classes of algorithms for the automatic generation of vascular beds: i) Fractal algorithms; and ii) Space-filling algorithms. The first class of algorithms can easily follow statistical models of the main morphometric measures (vessel radius, length, aspect ratio and bifurcation angle), regardless of the shape of the vascular territory^31–35^. The second class of algorithms allows one to generate a network of vessels inside a vascular territory defined in (2D or 3D) space^23,36–47^. A particular class of such space-filling algorithms is termed Constrained Constructive Optimisation (CCO) because the sequential generation of the vessel network is driven by the constrained minimisation of a cost function. Recently, methods for sprouting angiogenesis have also been proposed to study the angiogenic process involved in the formation of new blood vessels^48^.

In particular, the family of CCO algorithms addresses different types of study involving the influence of anatomical variability^44,49–53^, bifurcation asymmetries^42,54,55^, fractal properties^56^, staged-growth^46,57^, shear stress distribution^58,59^, among others^43,60–62^. Variants have also been proposed either to recreate more complex vascular networks in hollow organs^23,43,44,46,63,64^, or to speed-up the construction of such networks^46,65^. More recently, cases of territories supplied by multiple inlet vessels were the focus of attempts to tackle more realistic scenarios. In Blanco et al.^45^, Ii et al.^46^ and Di Gregorio et al.^47^, partitioning of a territory into subdomains was proposed so that the CCO algorithm could independently be applied to vascularise non-overlapping subdomains. Meanwhile, Jaquet et al.^43,66^ proposed to solve concurrency by assigning a relative flow quota for each input, while those who temporally exceed their quota are put on hold. Additionally, the latest works focused on describing whole-organ microvasculature^46,67^ defining multiple steps to generate a physiologically accurate tree. Each strategy defines different ad-hoc steps depending on the specific organ. In this work, we propose a staged growth strategy generalising the definition of such steps for the vascularisation of different tissues.

Some contributions have reported results of blood flow simulations performed with vascular networks reconstructed from images of animal models^25,68,69^. Similarly, the use of automatically generated networks in practice has mainly been restricted to academic cases or specific organs with a simplified description of the perfusion, because of intrinsic limitations in the generation procedure. For example, the use of fractal networks to assess closure boundary conditions in hemodynamic simulations^70,71^, or the use of CCO networks to predict the arteriolar pressure in the brain^72^. More detailed generated networks have been proposed for heart^43,47,66^ and brain^46,67^ following a methodology specific for the organ of interest.

In this work, we extend the capabilities of the traditional CCO algorithm, aiming to develop a general-purpose versatile approach for the generation of vascular networks in complex geometrical domains and, also, enhance the modelling capabilities to encode architectural features and constraints in the derived anatomical description. One example is the possibility of automatically generating vessels of networks either from scratch or from already existing networks of vessels. In fact, generation of networks from existing data (possibly acquired from medical images) is extremely important in providing a viable solution for the (still open) problem of setting patient-specific boundary conditions in hemodynamics. If the domain to be vascularised is known, and the larger vessels that supply such a territory are known, then it is possible to provide an anatomically consistent patient-specific completion of the vascular network. Moreover, if we consider studies employing the framework provided by the Anatomically Detailed Arterial Network (ADAN) model^73,74^, it is also possible to foresee *in silico* models of human physiology with an unprecedented level of vascular detail, by including arterioles and capillaries specifically tailored for each vascular territory across the whole model, making possible the coupling with organ-specific physiology.

Specifically, we propose an aDaptive CCO (DCCO) algorithm that can be used to vascularise empty regions of space as well as to fill pre-existing vascularised domains with additional vessels. The method is termed as adaptive because it provides new features to change the behaviour of the CCO –through different optimality criteria, vessel bifurcation sites, geometric constraints– across the filling procedure. This is accomplished by establishing staged hierarchies and constraints along the network generation process, being driven by possibly adaptive optimisation criteria. This flexibility in the setting of constraints and the cost function enables one to establish a connection between the vascularisation processes and underlying biological mechanisms. In fact, while the vascularisation of a certain organ from scratch can be viewed as a global architectural process, the addition of small vessels to an already highly vascularised region could be driven by models of angiogenic stimuli, for which optimisation criteria differ.

We test the DCCO algorithm for the generation of vascular networks in academic scenarios with simple domains and also in realistic scenarios involving multi-stage vascular outgrowth, starting from existing vascular networks taken from detailed human models^74^ in anatomically representative territories of the human body. For the various examples discussed here, we report the statistical properties of the circulatory networks obtained from the applied vascularisation processes.

## Methods

In this section, we introduce the standard CCO techniques followed by our extended approach as a set of new features and modelling tools. "Constrained constructive optimisation" section describes the standard CCO technique, main notation and optimisation strategy used during the construction process. "Staged growth" section presents our core contribution: the modelling concept of an elemental behavioural unit to define, in a sequential manner, phases of vascular development called growing stages. "Distribution, transport and perforator vessels" section defines types of vessels featuring different capabilities to communicate, perforate or vascularise domains. "Multi-criteria growth" section proposes an alternative cost function based on sprouting phenomena that delivers a balanced tree growth for domains with multiple inputs. "Blood flow distribution" section presents strategies to impose a heterogeneous blood flow distribution across the domain of perfusion, and to model outflow constrains in a given set of vessels. "Vascular completion" section introduces further extensions to consider a given vascular tree as the initial condition prescribing how its vessels will be optimised during the DCCO process. Lastly, we detail other geometrical extensions in "Other extensions" section.

### Constrained constructive optimisation

CCO methods provide an algorithmic approach for the construction of a binary vascular tree through the minimisation of a biologically-inspired cost functional. From early works in the field (*e.g.* Schreiner et al.^36,49^), the criterion was engineered to minimise the biological infrastructure and energy expenditure for blood supply to the surrounding tissues. In this case, the optimisation problem for the vascularisation of a territory $\Omega$ is stated as follows: find the vascular tree ${\mathscr {T}} = \{v_i=(r_i,x^p_i,x^d_i), i = 1,\ldots ,N \}$, within the set of admissible vascular trees ${\mathsf {T}}$, which fills the domain $\Omega$ with prescribed proximal position $x^p_1$, radius $r_1$ and inflow *Q* such that

1 $$\begin{aligned} {\mathscr {T}}&= \mathop {{{\,\mathrm{arg\,min}\,}}}\limits _{{\mathscr {T}}^*\in {\mathsf {T}}} {\mathscr {F}}({\mathscr {T}}^*), \end{aligned}$$

2 $$\begin{aligned} {\mathscr {F}}({\mathscr {T}})&=\sum _{i=1}^N l_i \pi r_i^2, \end{aligned}$$

where $r_i$, $l_i = \Vert x^d_i - x^p_i \Vert$, $x^p_i$ and $x^d_i$ are the radius, length, proximal and distal positions of the *i*-th vessel of the tree ${\mathscr {T}}$ and *N* is the total number of vessels of ${\mathscr {T}}$. The set ${\mathsf {T}}$ shapes the construction of the network, as it presents the constraints to be satisfied by the vessels and, also, the boundary conditions prescribed on the physical model. In this regard, two examples of simple boundary conditions that can be prescribed to a vascular tree are: (i) a given homogeneous outflow $q_f$ at all terminal vessels, or (ii) a pressure drop $\Delta p$ with respect to the reference pressure of the proximal vessel.

#### Geometrical constraints

Geometrical constraints arise to make the algorithmic vascularisation procedure compliant with observational data regarding the structural characteristics of vascular networks. To formally state these constraints let us define $v_p, v_{s1} \text { and } v_{s2}$ as the parent and daughter vessels at a particular bifurcation. The relation among vessel radii at a bifurcation is constrained to follow Murray’s law^75,76^

3 $$\begin{aligned} r_p^\gamma = r_{s1}^\gamma + r_{s2}^\gamma , \end{aligned}$$

where $\gamma$ is the power-law coefficient. For terminal vessels, the radius ratio between post-bifurcation vessels must satisfy the following inequality

4 $$\begin{aligned} \dfrac{\min (r_{s1},r_{s2})}{\max (r_{s1},r_{s2})} > \delta , \end{aligned}$$

where $\delta$ is the symmetry ratio parameter^37^; and the aspect ratio of each vessel is constrained to be

5 $$\begin{aligned} \dfrac{l_i}{r_i} > 2. \end{aligned}$$

Also, ${\mathscr {T}}$ is a dichotomously branching (binary) system of straight cylindrical segments without intersections (no physical inter-penetration).

#### Hemodynamics model

To ensure a specific pressure or flow at the tree terminals, a compartmental steady state flow model for Newtonian blood flowing into straight cylindrical vessels is adopted. In such a low Reynolds number model, inertia and compliance are neglected, and the blood flow is completely dominated by viscous effects. Hence, pressure drop at each vessel $v_i$ is characterised by

6 $$\begin{aligned} \Delta p_i = R_i \, q_i, \end{aligned}$$

where $R_i$ and $q_i$ are the vessel resistance and flow rate across $v_i$, respectively. At pre-arteriole and arteriolar levels (diameter $\approx$ 10–1000 $\upmu$m), resistance is assumed to follow Poiseuille’s law

7 $$\begin{aligned} R_i = \frac{8 \, \eta (r_i) \, l_i}{ \pi \, r_i^4}, \end{aligned}$$

with the fluid viscosity $\eta$ accounting for the Fȧhræus–Lindqvist effect^77^, as follows

8 $$\begin{aligned} \eta (r_i)&= 1.125 \left( \kappa + \kappa ^2 ( 6 \exp (-170 \, r_i) - 2.44 \exp \left( -8.09 \, r_i^{0.64}\right) + 2.2 \right) \Big ), \end{aligned}$$

9 $$\kappa = \left( \dfrac{r_i}{r_i-5.5\text {e}{-4}}\right) ^2,$$

where $r_i$ is the vessel radius in millimetres.

More complex models for viscosity –*e.g.* accounting for plasma skimming effect and, consequently, variable hematocrit discharge^42,67^– can be straightforwardly implemented with the proposed strategy by modifying the viscosity update (see Algorithm 1, line 5) with the chosen viscosity model.

#### Optimisation algorithm

A vascular tree is generated by incrementally adding vessels one by one. After the addition of each vessel, the vascular tree is remodelled to meet the geometric and hemodynamic restrictions. In that manner, the tree optimisation problem (2) is approximated by a sequence of per-vessel optimisation problems in a set of admissible vessels ${\mathsf {V}}$,

10 $$\begin{aligned} & v_{\text {new}} = \mathop {{{\,\mathrm{arg\,min}\,}}}\limits _{v\in {\mathsf {V}}} {\mathscr {F}}_{\text {vol}}(v) ,\\ &{\mathscr {F}}_{\text {vol}}(v) = {\mathscr {F}}({\mathscr {T}}^*({\mathscr {T}},v)) - {\mathscr {F}}({\mathscr {T}}), \end{aligned}$$

where ${\mathscr {T}}^*({\mathscr {T}},v)$ is the tree obtained after adding *v* to ${\mathscr {T}}$ and reshaping it to satisfy the constraints posed in ${\mathsf {T}}$, and inherited by ${\mathsf {V}}$. In contrast to other CCO strategies^37,56^, we fix the radius of the vascular inputs and the domain size, since such an approach offers advantages for the extensions proposed in the following sections. To generate a new vessel, a terminal point is randomly created following a uniform distribution across the entire domain and is then attached to ${\mathscr {T}}$ at a locus such that it satisfies the geometrical restrictions presented in “Geometrical constraints” section. Notice that more complex vascularisation patterns can be modelled by modifying the hypothesis of uniformity in the definition of terminal location, as discussed in "Blood flow distribution" section.

It is noteworthy that random (uniform) generation of terminal points may be prone to imbalance for a certain region of the domain, due to a biased early growth of the tree. Assuming a convex domain, balanced trees are more efficient in terms of tree volume and energy expenditure. Because the early steps of the tree generation determine the main topological features of the tree, it is the key to promoting a homogeneous dispersion of such points (favouring balanced trees). Thus, a minimum distance constraint between a new terminal point, say *x*, and the current tree ${\mathscr {T}}$ is used to reject points close to the pre-existing tree in the following manner

11 $$\begin{aligned} |x - x_t |< l_{\text {lim}}, \end{aligned}$$

where $x_t$ is the point in ${\mathscr {T}}$, closest to *x* and $l_{\text {min}}$ is computed as

12 $$l_{{{\text{min}}}} = l_{c} {\text{ }}\sqrt[D]{{\frac{\nu }{{N_{T} + 1}}}},$$

where $N_T$ is the number of terminal vessels already in $\mathscr {T}$; $l_c$ and *D* are, respectively, the characteristic length and dimension of $\Omega$; and $\nu$ is a tuning parameter whose role is to ensure that the tree provides uniform coverage in slender domains. The characteristic length represents the expected perfusion radius of the terminals at the current generation step, turning $l_{\text {min}}$ into the minimum distance to avoid overlapped perfusion from different terminals. For this reason, it is taken as

13 $$l_{c} = \left\{ {\begin{array}{*{20}l} {\sqrt {\frac{{\int_{\Omega } d A}}{\pi }} } \hfill & {\quad {\text{for}}\,\,\,D = 2,} \hfill \\ {{\text{ }}\sqrt[3]{{\frac{{3\int_{\Omega } d V}}{{4{\mkern 1mu} \pi }}}}} \hfill & {\quad {\text{for}}\,\,\,\,D = 3.} \hfill \\ \end{array} } \right.$$

Notice that, for slender domains, such approximations of $l_c$ underestimate the perfusion distance of the terminals, since the perfusion will be concentrated towards the dominant axis reaching larger distances. This underestimation effect is expected to be reduced as the tree grows, and the perfusion per terminal becomes small enough to fit into the shorter axis of the domain. Because of these issues, the parameter $\nu$ has been added to the standard definition (see (12)), which has a considerable effect for small values of $N_T$, and fades away as $N_T$ increases.

In the definition of $l_{\text {lim}}$ in (12), the perfusion area is also overestimated because it is assumed to occur along the whole tree and not just in the terminals. Thus, a correction step by a factor of $f_r$, *i.e.*,

14 $$\begin{aligned} l_{\text {lim}} = f_r \, l_{\text {lim}}, \quad { 0< f_r < 1}, \end{aligned}$$

is performed in case $N_{\text {fail}}$ consecutive attempts to generate $x_t$ fail to satisfy (11).

**Figure 1:**
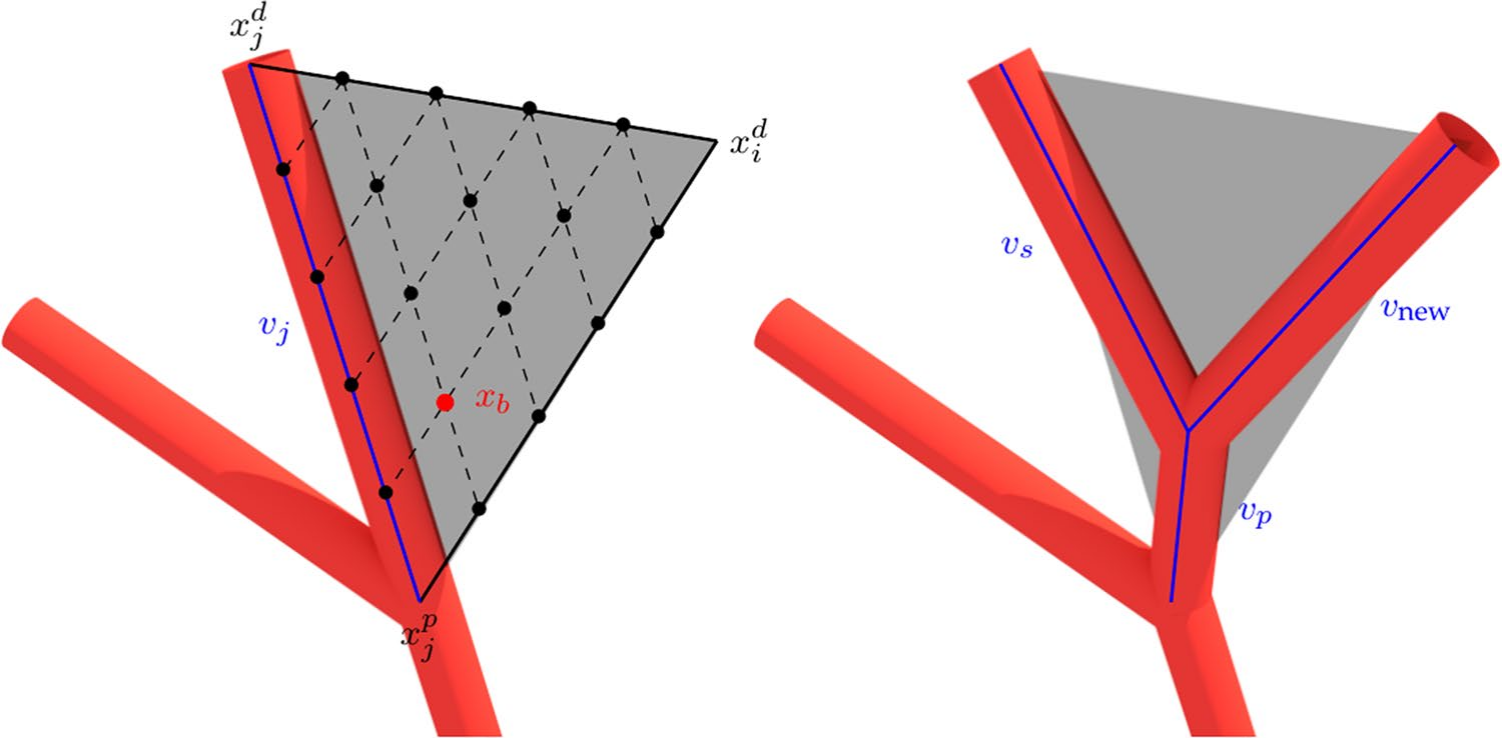
Bifurcation optimisation strategy for $\Delta v = 6$: (left) discretised domain to look for the optimal bifurcation point $x_b$ between vessel $v_j$ and the candidate terminal point $x^d_i$; (right) new tree structure for optimal bifurcation point $x_b$ replacing vessel $v_j$ by vessels $v_{\text {p}}$, $v_{\text {s}}$ and $v_{\text {new}}$ in ${\mathscr {T}}$. All black dots in the left image denote the set of potential bifurcation points. Images generated with Blender v2.8 available at https://www.blender.org/.

Once a new plausible terminal point has been obtained, we must find the connection point in ${\mathscr {T}}$ such that it optimises (10) while satisfying the geometrical and hemodynamic constraints (*i.e.* such that the vessel belongs to ${\mathsf {V}}$). Note that as we connect the terminal $x^d_i$ to a vessel $v_j$ in the tree, the later is replaced by three new vessels $v_{\text {p}}$, $v_{\text {s}}$ and $v_{\text {new}}$, as shown in Fig. 1. To assess the best junction site, the cost function ${\mathscr {F}}$ is computed for all potential connections between the new terminal point and each vessel $v_j$, within a neighbourhood radius of $f_n \, l_c$. Then the connection with minimum cost is effectively achieved. For each connection between the new terminal $x^d_i$ and $v_j$, we search for the bifurcation point $x_b$ within the triangle defined by coordinates $(x^d_i,x^p_j,x^d_j)$ that minimises ${\mathscr {F}}$. This optimisation is performed heuristically by discretising the domain of the triangle into a set ${\mathscr {J}}$ of $P_{\text {opt}} = \sum \nolimits _{i=1}^{\Delta v} i - 3$ points (where $\Delta v$ is the number of points used for the discretisation of a triangle side) as shown in Fig. 1, and choosing by brute force the bifurcation site in this set with minimum $\mathscr {F}$ value. If any of the bifurcation vessels (*i.e.*, $v_{\text {p}}$, $v_{\text {s}}$ and $v_{\text {new}}$) intersects another vessel, leaves the domain, or violates any geometrical constraint, then the bifurcation point is discarded as a candidate solution. If all points of the discretisation are excluded, then no optimal point exists for that connection. To compute the cost function $\mathscr {F}$ for each bifurcation trial point, we temporarily exclude $v_j$ from ${\mathscr {T}}$, add the corresponding bifurcation vessels $v_{\text {p}}$, $v_{\text {s}}$ and $v_{\text {new}}$, and update the radii of the network ${\mathscr {T}}$. Since the viscosity depends upon the vessel radius, we use a slight variation of the scheme presented in Karch et al.^51^ to update the tree radii, based on a fixed-point scheme, as introduced in Algorithm 1. 
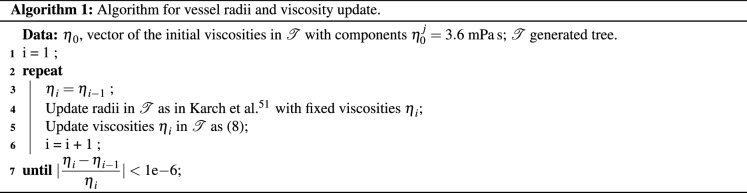

Taking into account all the descriptions above, we formulate a procedure to generate the vascular tree ${\mathscr {T}}$ (see Algorithm 2). First, we search for a first terminal to create a root segment that satisfies (5) with an acceptable perfusion length (line 1 to 6). Then, we incrementally generate a new terminal point per iteration in the following three steps: (i) search for a terminal point that complies with the minimum distance to $\mathscr {T}$ to avoid perfusion overlap (line 10 to 14); (ii) search in a local set of neighbouring vessels for the best connection site (*i.e.*, we search for the vessel $v_j \in {\mathscr {T}}_n$ that yields the bifurcation with the lowest value of ${\mathscr {F}}$ meeting the geometrical constraints); (iii) if one or more bifurcation sites are valid we add such a terminal to the tree ${\mathscr {T}}$ using the bifurcation site that yields the lowest value of ${\mathscr {F}}$ and then update the vessel radii in the entire tree (lines 27 and 28), otherwise we go back to step (i). 
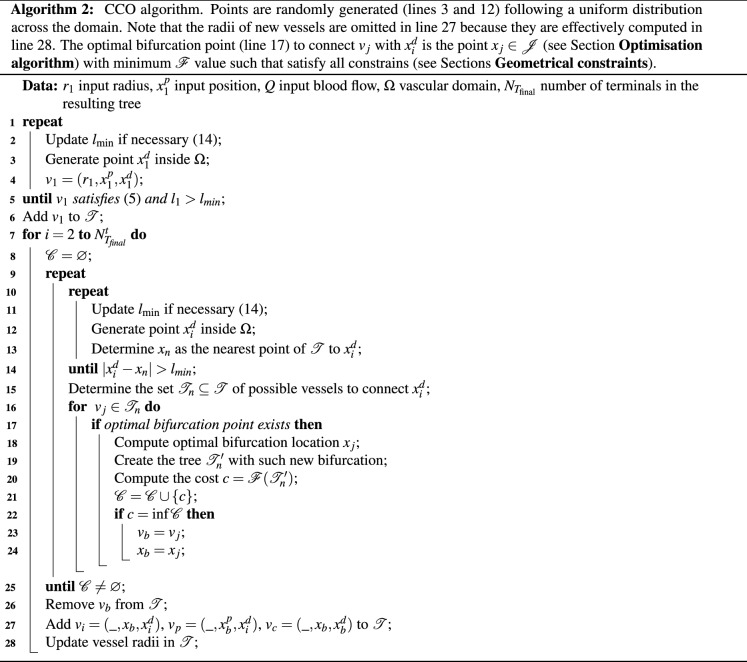


### Staged growth

Previously, Karch et al. introduced the concept of staged growth using a time dependent strategy^57^, where the CCO domain grows as a function of an elapsing pseudo-time which evolves as terminals are added to the tree. Interestingly, dominant directions of the domain growth imprint an anisotropic pattern in the vascular tree. In our proposal, the concept of stage is rather different. Here the stage is a static problem where a tree grows by adding a given number of new terminals. Also, each of them may involve a different domain (or topological variations of one of the domains), different geometrical or hemodynamic parameters and different growing conditions (growth of different branches of pre-existing trees, or different growing rules—see "Multi-criteria growth" and "Blood flow distribution" sections) triggered by embryonic, remodelling and/or environmental stimuli. This approach allows us to model specific stages to mimic different developmental processes, tissue structures, or vascular territories featuring common network architectures.

In the proposed approach, the growth of the vascular tree is executed in a finite sequence of stages ${\mathscr {S}}_1, {\mathscr {S}}_2, \ldots , {\mathscr {S}}_M$. Formally, a stage is defined by a set ${\mathscr {S}}_t= \{ \Omega , {\mathscr {P}}_{\text {geo}}, \mathscr {P}_{\text {opt}}, {\mathscr {T}}_{\text {init}}, N_{\text {T}_{\text {final}}} \}$ describing the problem conditions, where $\Omega$ is the vascular domain, ${\mathscr {P}}_{\text {geo}}=\{\gamma , \delta \}$ are the geometrical parameters, ${\mathscr {P}}_{\text {opt}}=\{\nu ,f_r,f_n,\Delta v\}$ are the optimisation parameters, ${\mathscr {T}}_{\text {init}}$ is the initial tree and $N_{\text {T}_{\text {final}}}$ is the number of new terminals to be generated. The initialisation tree $\mathscr {T}_{\text {init}}$ is the tree generated at previous stages or a subset of its vessels. The growth stage ${\mathscr {S}}_t$ is then performed by solving the optimisation problem presented in (2) using the Algorithm 3 (which employs the same strategy as Algorithm 2 for finding the optimal bifurcation points). As a result, each stage ${\mathscr {S}}_t$ generates a set of vessels, resulting in the stage tree $\mathscr {T}_t$, which is joined to the set of vessels in the global tree ${\mathscr {T}} = \bigcup \nolimits _{t=1}^{M} {\mathscr {T}}_t$. 
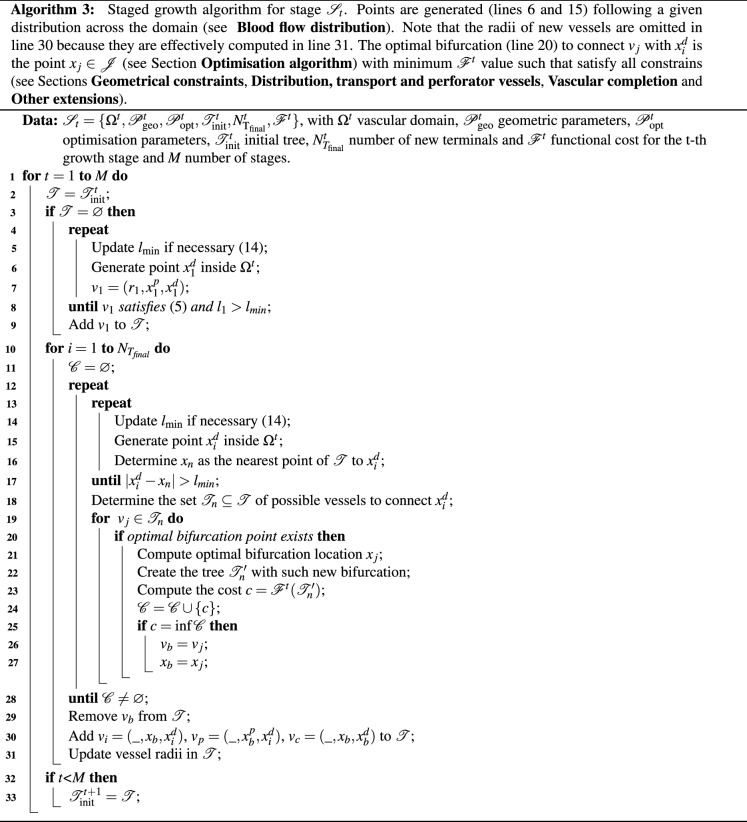


**Figure 2:**
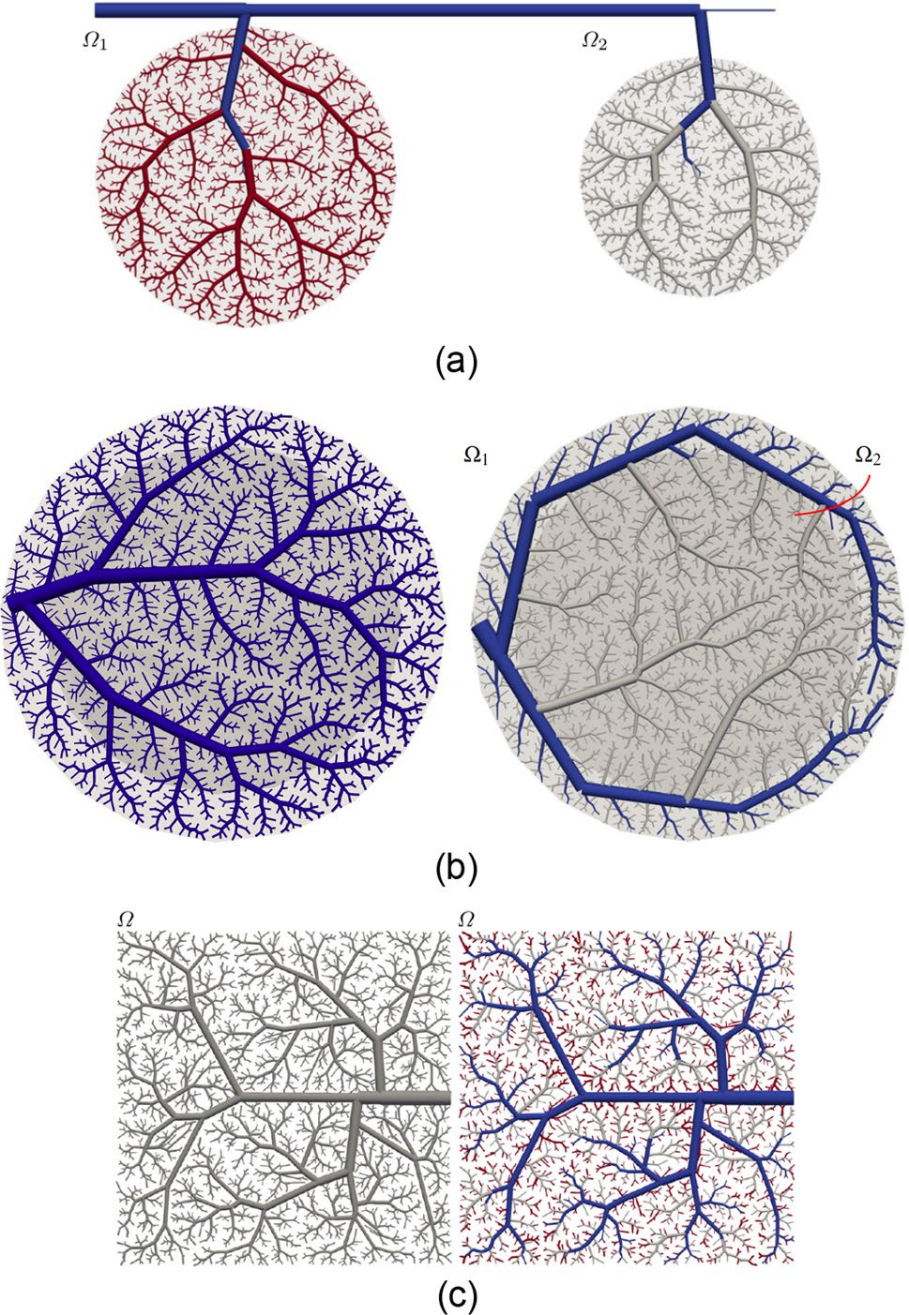
Different vascular architectures obtained using the staged growth paradigm. (**a**) Sequential vascular growth: blue vessels correspond to a pre-defined existing vascularisation ${\mathscr {T}}_0$, while red and grey ones correspond to trees ${\mathscr {T}}_1$ and ${\mathscr {T}}_2$, generated at stages ${\mathscr {S}}_1$ and ${\mathscr {S}}_2$. (**b**) Hierarchical vascular growth: (left) vascularisation obtained with a standard CCO algorithm with parameters ${\mathscr {P}}_{\text {geo}}$ and ${\mathscr {P}}_{\text {opt}}$; (right) the first stage of the network is ${\mathscr {T}}_1$, grown in the annulus subdomain $\Omega _1$ (light grey), while the second stage is ${\mathscr {T}}_2$, the inner circular subdomain $\Omega _2$ (dark grey). (**c**) Scale-specific vascular growth: (left) vascularisation obtained with a standard CCO algorithm with $\delta = 0.7$; (right) vascularisation using three stages with with $\delta = 0.7$, 0.5 and 0.2 for ${\mathscr {T}}_1$ (blue vessels), ${\mathscr {T}}_2$ (grey vessels) and ${\mathscr {T}}_3$ (red vessels), respectively. Images generated with ParaView version 5.4 available at https://www.paraview.org/.

To illustrate the capabilities of this new approach, the following scenarios are envisaged: (i) sequential vascular growth; (ii) hierarchical perfusion; and (iii) scale-specific vascularisation patterns. Consider in the following descriptions the schemes displayed in Fig. 2.

The following sections introduce new strategies that enhance the staged growth capabilities to attain highly customised vascular architectures.

#### Sequential vascular growth

This case embraces the simplest scenario in which a given domain is vascularised, then another domain is vascularised –with possibly different parameters–, and so on. For example, a pre-existing tree (denoted by ${\mathscr {T}}_0$, in blue) contains the inputs to two different vascular territories $\Omega _1$ and $\Omega _2$. Then, stages ${\mathscr {S}}_1=\{ \Omega _1, {\mathscr {P}}_{\text {geo}}, \mathscr {P}_{\text {opt}}, {\mathscr {T}}_0, 800\}$ and ${\mathscr {S}}_2=\{ \Omega _2, {\mathscr {P}}_{\text {geo}}, {\mathscr {P}}_{\text {opt}}, {\mathscr {T}}_1, 1200\}$ are executed with ${\mathscr {P}}_{\text {geo}} = \{\gamma =3,\delta =0\}$ and ${\mathscr {P}}_{\text {opt}}=\{ \nu =1.5, f_r=0.9, f_n=8, \Delta v=7\}$, generating the subtrees ${\mathscr {T}}_1$ (in grey) and ${\mathscr {T}}_2$ (in red) respectively.

#### Hierarchical vascular growth

In many organs across the human body, it is not unusual to find that the vascular network in a certain domain is responsible for the vascularisation of adjacent areas, resulting in a perfusion hierarchy. To model such a case, let us define two domains $\Omega _1$ and $\Omega _2$ (parent and sibling domains) where $\Omega _1$ is a ring-shaped domain and $\Omega _2$ is the circular domain enclosed by $\Omega _1$. Thus, the stages ${\mathscr {S}}_1=\{ \Omega _1, {\mathscr {P}}_{\text {geo}}, \mathscr {P}_{\text {opt}}, \varnothing , 50\}$ and ${\mathscr {S}}_2=\{ \Omega _1 \cup \Omega _2, {\mathscr {P}}_{\text {geo}}, {\mathscr {P}}_{\text {opt}}, \mathscr {T}_1, 1950\}$ are executed with ${\mathscr {P}}_{\text {geo}} = \{\gamma =3,\delta =0\}$ and ${\mathscr {P}}_{\text {opt}}=\{ \nu =1, f_r=0.9, f_n=8, \Delta v=7\}$, generating the subtrees ${\mathscr {T}}_1$ (in blue) and ${\mathscr {T}}_2$ (in grey), respectively. As a result, in the present example, the sibling domain is mainly perfused by the larger vessels in the parent domain featuring a hierarchical relationship.

#### Scale-specific vascular growth

As earlier periods during the development of a vascular network are related to bigger spatial scales in the final outcome, we can make use of the staged growth strategy to define scale-specific morphological patterns in the vascularisation process. In that case, several stages are performed in the same vascular domain $\Omega$ but with different geometrical constrains. Thus, the stages ${\mathscr {S}}_1=\{ \Omega , {\mathscr {P}}_{\text {geo}}^1, {\mathscr {P}}_{\text {opt}}, \varnothing , 50\}$, ${\mathscr {S}}_2=\{ \Omega , {\mathscr {P}}_{\text {geo}}^2, \mathscr {P}_{\text {opt}}, {\mathscr {T}}_1, 450\}$ and ${\mathscr {S}}_3=\{ \Omega , {\mathscr {P}}_{\text {geo}}^3, {\mathscr {P}}_{\text {opt}}, {\mathscr {T}}_2, 1500\}$ are executed with $\mathscr {P}_{\text {geo}}^1=\{\gamma =3,\delta =0.7\}$, $\mathscr {P}_{\text {geo}}^2=\{\gamma =3,\delta =0.5\}$, $\mathscr {P}_{\text {geo}}^3=\{\gamma =3,\delta =0.2\}$ and $\mathscr {P}_{\text {opt}}=\{ \nu =1.5, f_r=0.9, f_n=8, \Delta v=7\}$, generating the subtrees ${\mathscr {T}}_1$ (in blue), ${\mathscr {T}}_2$ (in grey) and ${\mathscr {T}}_3$ (in red), respectively. When comparing with a traditional CCO tree (Fig. 2c on the left) it is noted that the macroscale anisotropic characteristics are preserved by the first stage while the following ones generate isotropic networks at the smaller scales. This isotropy occurs because of the relaxation of the symmetry constraint (smaller values of $\delta$ allow larger vessels to bifurcate into small branches favouring coverage from local vessels instead of respecting the hierarchy imposed by vessel size).

### Distribution, transport and perforator vessels

Traditional CCO methods are focused on solving a local vascularisation problem where all generated vessels are embedded within the organ/tissue domain $\Omega$. That generates a constraint on certain vascularisation features such as the creation of perforator vessels from pre-existing networks (acquired from medical images or from anatomical atlases) or vascularisation between disjoint domains. To account for such possibilities, we propose to assign the vessels with a specific role, namely: distribution, transport or perforator. To present a proper definition of each role, let us define the domain $\Omega$, its complement $\Omega ^c$ (*i.e.* $x \in \Omega ^c \iff x \not \in \Omega$) and a vessel $v_j$ whose behaviour during the bifurcation process will be described for each role.

A *distribution vessel* is a vessel which is completely embedded within $\Omega$ and only bifurcates inside $\Omega$ as well. In the standard CCO algorithm all vessels play this role. A *transport vessel* is a vessel which is embedded and also bifurcates within the extended domain $\Omega \cup \Omega ^c$. Such vessels can be employed to communicate disjoint subdomains, where one subdomain vascularises the other one. Another usage of this role is the creation of redundant feeding networks from vessels in $\Omega ^c$, prior to the generation of the local vascular network in $\Omega$. A *perforator vessel* is a vessel which is embedded within $\Omega \cup \Omega ^c$ but only bifurcates inside $\Omega$. Such perforator vessels supply blood to the subdomain $\Omega$.

### Multi-criteria growth

**Figure 3:**
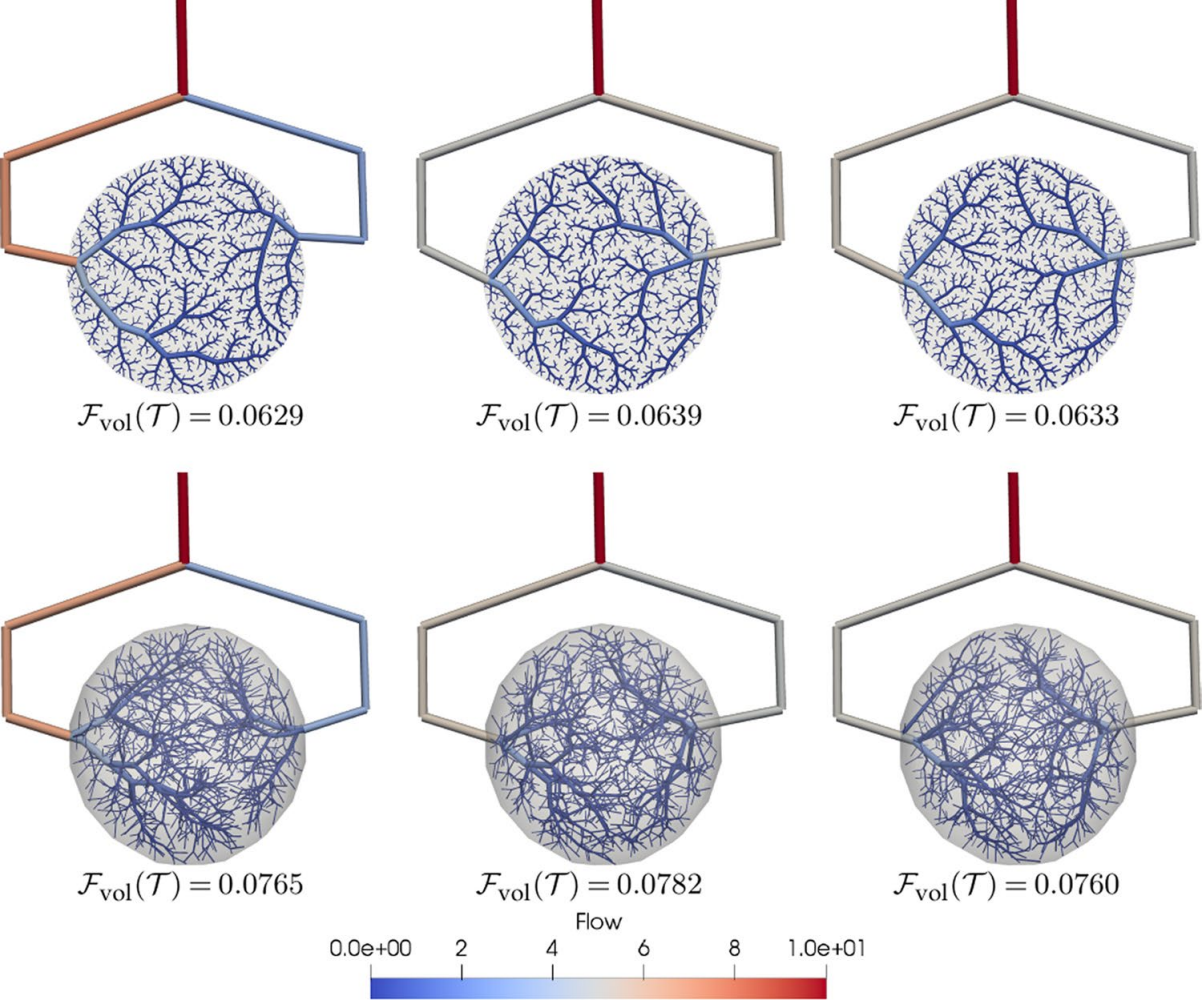
Concurrent vascularisation of circular and spherical domains with equiradial vessel inlets using: (left) volumetric cost criterion (standard CCO algorithm), ${\mathscr {S}}=\{ \Omega , \mathscr {P}_{\text {geo}}, {\mathscr {P}}_{\text {opt}}, {\mathscr {T}}, 1000, \mathscr {F}_{\text {vol}}\}$; (centre) sprouting cost criterion, ${\mathscr {S}}=\{ \Omega , {\mathscr {P}}_{\text {geo}}, {\mathscr {P}}_{\text {opt}}, {\mathscr {T}}, 1000, {\mathscr {F}}_{\text {sprout}}\}$; (right) 2-stage multi-criteria growing with ${\mathscr {S}}_1=\{ \Omega , {\mathscr {P}}_{\text {geo}}, {\mathscr {P}}_{\text {opt}},{\mathscr {T}}, 25, {\mathscr {F}}_{\text {sprout}}\}$ and ${\mathscr {S}}_2=\{ \Omega , {\mathscr {P}}_{\text {geo}}, \mathscr {P}_{\text {opt}}, {\mathscr {T}}, 975, {\mathscr {F}}_{\text {vol}}\}$. Parameters for central and right column cases are $c_v=0.5 \times 10^2, c_p= 0.5, c_d=1.0$ and $c_v=1.0 \times 10^4, c_p= 0.5, c_d=1.0$ for 2D and 3D cases, respectively, ${\mathscr {P}}_{\text {geo}} = \{\gamma =3,\delta =0\}$ and ${\mathscr {P}}_{\text {opt}}=\{ \nu =1.0, f_r=0.9, f_n=8, \Delta v=7\}$. Total vascular volume $F_{\text {vol}}({\mathscr {T}})$ is reported below each tree. Note that ${\mathscr {F}}_{\text {sprout}}$ preserves the initial balanced conditions of inlet vessel radii, while ${\mathscr {F}}_{\text {vol}}$ tends to benefit one inlet over the other. Images generated with ParaView version 5.4 available at https://www.paraview.org/.

The volumetric cost function presented in (10) leads to reasonable results when vascular volume and energy expenditure optimisation are the main contributing factors towards angiogenesis. However, depending on the underlying organ or vascular territory, other factors such as blood supply redundancy or concurrent perfusion between branches must be considered as well. For example, in Fig. 3, the criterion given by (10) induces a more prominent growth of one of the branches over the other.

Taking advantage of the staged growth, a different optimisation criterion can be employed for each specific stage. In such case, the definition of a stage is extended as ${\mathscr {S}}_t= \{ \Omega , {\mathscr {P}}_{\text {geo}}, {\mathscr {P}}_{\text {opt}}, \mathscr {T}_{\text {init}}, N_{\text {T}_{\text {final}}}, {\mathscr {F}} \}$, that is, we include the cost function as an additional feature of each stage. To cope with concurrent vascularisation issues, a novel optimisation criterion, referred to as sprouting cost function, is proposed which extends the previous model to account for energy expenditure due to vessel-wall degradation of the parent vessel and growth-signal diffusion. Then the sprouting cost of adding a new vessel $v_{\text {new}}$ is formulated as

15 $$\begin{aligned}{}&v_{\rm new} \mathop {\arg \,\min }\limits_{{v \in V}} {\mathcal{F}}_{\rm sprout} (v),\\& {\mathcal{F}}_{\rm sprout} (v) = \left[ {c_{v} \frac{{{\mathcal{F}}_{\rm vol} (v)}}{{V_{\rm ref} }} + c_{p} \frac{{r_{p} }}{{r_{\rm ref} }} + c_{d} \left( {\frac{{||x^{d} - x^{p} ||}}{{l_{\rm ref} }}} \right)^{2} } \right] ,\\&V_{\rm ref} \quad l_{\rm ref} = \sqrt[3]{{\frac{{3V_{{{{\rm ref}}}} }}{{4\pi }}}} \quad r_{\rm ref} = r_{1} , \end{aligned}$$

where we must recall that a vessel is characterised by $v=(r,x^p,x^d)$, ${\mathscr {F}}_{\text {vol}}$ is the volume variation due to the addition of *v* to ${\mathscr {T}}$ (see (10)) and $r_p$ is the radius of the parent vessel of *v*. The terms are scaled by the mixture coefficients $c_v, c_p, c_d$ and are normalised by means of quantities $V_{\text {ref}}$, $l_{\text {ref}}$ and $r_{\text {ref}}$ which are the vascularised volume, its equivalent radius, and the root vessel radius, respectively. The first term is analogous to (2), and if this term is dominant we are led to the vascular patterns seen before. The second term accounts for the cost behind the degradation of the parent vessel wall. In short, this degradation mechanism is responsible for exposing endothelial cells, and for triggering the sprouting process. The cost is proportional to the thickness of the vessel wall which, in turn, is assumed to be linearly related to the vessel radius. The third term models the cost for growth-signal diffusion from the new terminal point to the closest point of the parent vessel described by Fick’s second law.

The proposed formulation allows us to weigh contributions related to growth factor expression and the parent vessel remodelling. Particularly, the degradation term favours smaller parent vessels while the growth-signal diffusion term favours closer parent vessels to the new terminal point. By selecting appropriate values of the weighing parameters, we obtain a balanced vascularisation of the tissue in term of its inputs (see Fig. 3 where the use of $\mathscr {F}_{\text {sprout}}$ results in an even flow supply from both lateral inputs of the circular and spherical domains). Note that the cost function ${\mathscr {F}}_{\text {sprout}}$ presents less physiological branching patterns, and may also produce detrimental effects for small vessels in terms of volume cost (central column) because of the trade-off between volumetric optimisation and sprouting energy expenditure (terms 2 and 3 in (15)). These unfavourable effects can be mitigated by using two stages: $\mathscr {S}_1$ optimising sprouting cost and ${\mathscr {S}}_2$ optimising volume cost. Thus, a balanced and efficient tree, in terms of vascular volume, is generated (right column). Another important aspect to take into consideration is that the optimisation process for both functional costs are carried out by a heuristic approach, which does not necessarily reach the global minimum. Because of that, we can observe that for the 3D case the volumetric cost criterion render a vasculature with larger volume than its 2-stage counterpart.

### Blood flow distribution

Most organs in the human body present a heterogeneous blood flow distribution. The particular distribution pattern depends not only upon constitutive and functional characteristics of the underlying tissue such as cell metabolism, density and phenotype, but also upon the chronological record of developmental events. Thus, the generation of physiologically compatible vascular trees must be endowed with a mechanism to prescribe distribution criteria in the vascularised domain which can mirror prior knowledge about that specific territory.

As previously stated in "Constrained constructive optimisation" section, the vascular tree is constrained to supply a blood flow *Q* across a certain domain $\Omega$ through *M* terminal vessels. In that setting, all terminals are assumed to have the same outflow $q_f$ with a distal position determined by the spatial probability distribution function $p(x) \sim {\mathscr {D}}$. Note that if $M \rightarrow \infty$, the blood flow distribution in the domain is a scaled version of ${\mathscr {D}}$, and in fact the blood flow of a sub-region $\omega$ is given by

16 $$\begin{aligned} \int _{\omega } q(x) \, \text {d}\omega = \frac{Q}{\int _\Omega p(x) \; \text {d}\Omega }\int _{\omega } p(x) \; \text {d} \omega \end{aligned}$$

**Figure 4:**
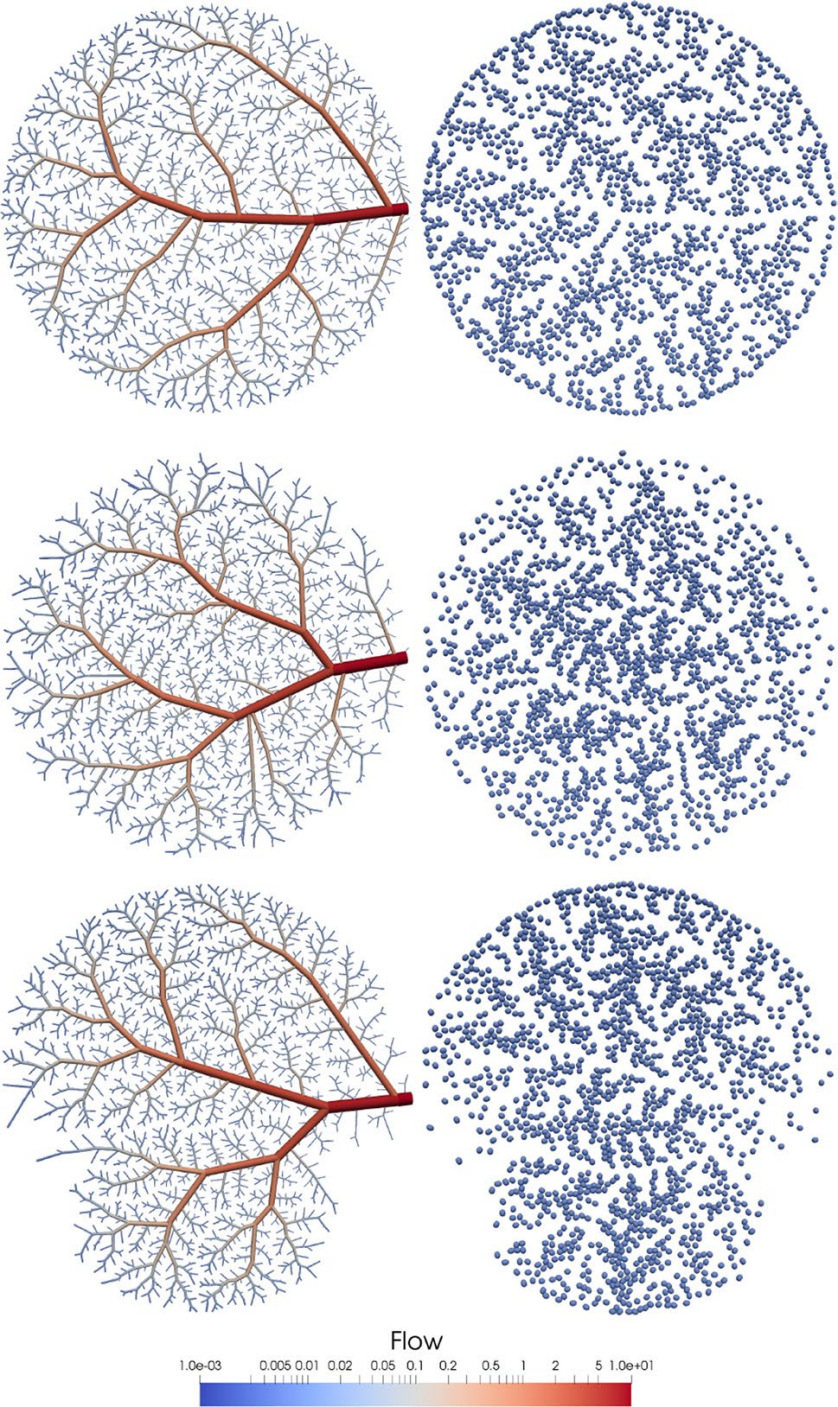
Flow delivery using different probability distribution functions for $x_i^d$ generation: (first row) uniform distribution (standard CCO algorithm); (second row) Gaussian distribution $x_i^d \sim \mathscr {N}(\mu ,\sigma ^2)$ with $\mu = (0,0)$ and $\sigma =(0.25,0.25)$; and (third row) Gaussian mixture of 2 distributions $x_i^d \sim 0.5 \, {\mathscr {N}}(\mu _1,\sigma _1^2) + 0.5 \, {\mathscr {N}}(\mu _2,\sigma _2^2)$ with $\mu _1 = (0,0.5)$, $\sigma _1=(0.25,0.25)$, $\mu _2 = (0,-0.5)$ and $\sigma _2=(0.10,0.10)$. First column presents the generated tree while second column outlines the distribution of the tree terminals that are sampled from the probability distribution. The domain $\Omega$ is a circle of radius 1 centred at (0, 0) with positive axes pointing towards the right and upper directions. Images generated with ParaView version 5.4 available at https://www.paraview.org/.

Thus, the distribution ${\mathscr {D}}$ shapes the blood flow delivery across the domain, and may vary according to the structure and functionality of the underlying tissues. Then, we can prescribe a desired blood flow distribution over the domain by using it as the distribution to generate new terminals. In Fig. 4, three different distributions are imposed over the domain: (i) a traditional uniform distribution; (ii) a Gaussian distribution; and (iii) a Gaussian mixture distribution. In these cases, the regions with higher probability feature higher concentration of terminal points. The centred Gaussian distribution in case (ii), shows a heavily vascularised centre and a sparser vascularisation near the boundaries. The mixture Gaussian distribution, shows that regions with low probabilities are vascularised further ahead during the growing process.

**Figure 5:**
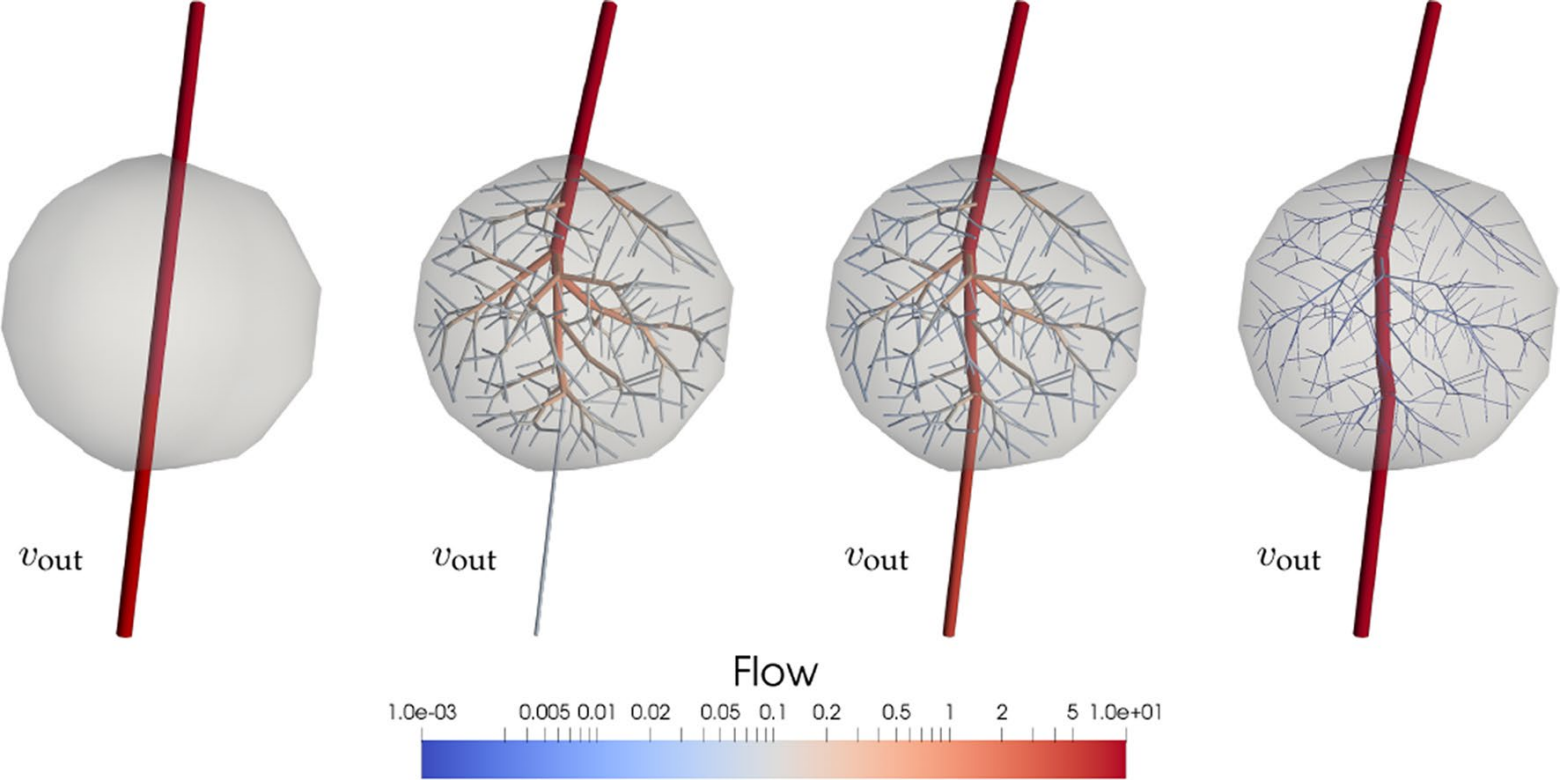
Flow delivery in the presence of an outlet vessel $v_{\text {out}}$ with a prescribed outflow: (from left to right) tree initialisation, flow to be distributed is *Q*; $v_{\text {out}}$ flow is unconstrained, i.e., outlet carries the same flow than terminals inside the domain; $v_{\text {out}}$ flow is constrained to $0.5 \, Q$; and $v_{\text {out}}$ flow is constrained to $0.99 \, Q$. Images generated with ParaView version 5.4 available at https://www.paraview.org/.

Another important physiological aspect is that not the entire blood inflow is to be distributed in the domain. Some vessels may divert part of the flow to adjacent vascular territories. To model this behaviour, the vascular tree is bestowed with a certain number of distal arteries with prescribed outflows, denoted as $q^{\text {out}}_i$. The constrained flow through these vessels is imposed as a fraction of the inflow *Q*. In Fig. 5, it is shown how this constraint affects the vessel radii in the generated vasculature. In particular, as the output flow increases, so does the resistance of the vascular network, which is reflected through an overall decrease in the vessel radii by a factor of $\sqrt[4]{{\frac{{R_{\rm new} }}{{R_{\rm old} }}}}$.

### Vascular completion

From medical imaging or atlas models, a proximal (or large scale) description of the vasculature can be available for different organs or tissues. Once the geometrical description is modelled as a *N*-ary tree (see "Generalised tree structure" section), the proposed DCCO method can blend this information in the constrained optimisation process forcing a specific branching behaviour for each of the pre-existing vessels. We propose four different branching behaviours: (i) non-branching; (ii) distal; (iii) fixed; and (iv) versatile. Each of these options will determine if the vessel is eligible to become a parent vessel of new terminals (see line 13 of Algorithm 3) and, if eligible, the set of potential bifurcation points (see Fig. 1) that are allowed to perform the optimisation procedure.

- *Non-branching vessel* These vessels are not eligible as parent vessel for new terminals during the optimisation process.
- *Distal vessel* These vessels are eligible as parent vessel but only its distal point can be regarded as a potential bifurcation point.
- *Fixed vessel* These vessels are eligible as parent vessel but the location of the potential bifurcation point can only be placed along the straight line joining $x_j^p$ and $x_j^d$.
- *Versatile vessel* These vessels are eligible as parent vessel and define the same set of potential bifurcation points as the vessels generated by the version of the DCCO method described in the previous sections.

### Other extensions

Here, we summarise two other minor contributions of the proposed DCCO approach.

#### Bifurcation angle constraint

Two different constraints are proposed: (i) the bifurcation angle $\theta$ and (ii) the opening angle $\phi$. The bifurcation angle is defined as the angle $\theta _{v_s,v_{\text {new}}}$ between vessels $v_s$ and $v_{\text {new}}$. Thus, we remove the bifurcation points from the optimisation process that do not satisfy the following constraint

17 $$\begin{aligned} \theta _{\text {min}}< \theta _{v_s,v_{\text {new}}} < \theta _{\text {max}}, \quad \text {with}\quad \theta _{v_s,v_{\text {new}}} = \arccos \frac{\langle x^d_j - x_b, x^d_i - x_b\rangle }{ l_{v_s} \, l_{v_{\text {new}}}} \end{aligned}$$

where $\langle \cdot , \cdot \rangle$ is the conventional inner product in the Euclidean space, and $v_s,v_{\text {new}},x^d_j, x_b$ and $x^d_i$ are defined in Fig. 1. The opening angle $\phi$ is the angle between $v_{\text {new}}$ and the plane defined by $v_s$ and $v_p$. As before, we remove the bifurcation candidates that do not satisfy

18 $$\begin{aligned} \phi _{\text {min}}< \phi < \phi _{\text {max}}, \quad \text {with}\quad \phi = 90^{\circ } - \arccos \frac{|\langle n, x^d_i - x_b\rangle |}{ \Vert n \Vert \, l_{v_{\text {new}}}} \end{aligned}$$

where $n = (x^d_j - x_b) \times (x^p_j - x_b)$ is the normal vector to the bifurcation plane, computed as the cross product between the vectors that define the direction of $v_p$ and $v_s$.

#### Generalised tree structure

In DCCO, ${\mathscr {T}}$ is extended to model a *N*-ary tree where an arbitrary number of vessels can branch at each junction. This feature enhances the capabilities to represent pre-existing vascular descriptions from anatomic atlases or medical images (see "Applications" section) as initial condition of our optimisation process. Vessels with high tortuosity are quite common in the cardiovascular system, which are inaccurately represented by a single straight segment but can easily be represented through several unary branches. Infrequent cases such as trifurcations (or higher branching multiplicity) can also be straightforwardly modelled with this approach.

## Applications

To illustrate the capabilities of the proposed method, we generate the arterial vasculature of different tissues designed to match qualitative and quantitative features with the domain knowledge. For each case, we detail the stages and the rationale applied to reproduce the vascular patterns at the different regions or scales, aiming at a pragmatic understanding of the techniques proposed in "Methods" section.

In practice, an initial vascular structure could be available from medical images. A similar scenario here will be addressed by growing vascular networks from pre-existing vascular structures from the ADAN model^74^. Available data include anatomical position, vessel radii and blood flow of the main arteries which will be used as the initial tree ${\mathscr {T}}_0$ for the DCCO method.

**Figure 6:**
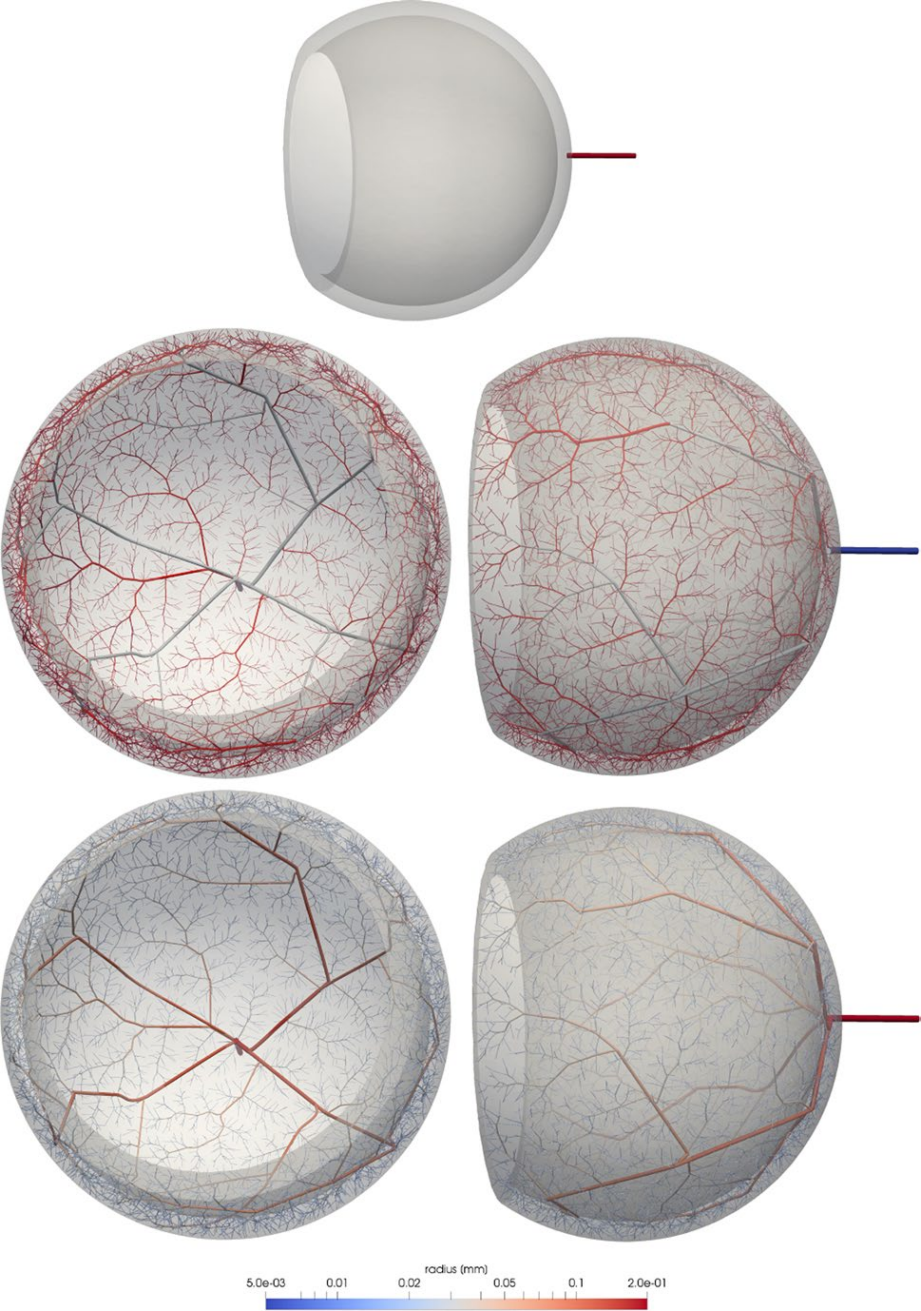
Inner retinal vasculature generated by a two-staged growth process: (first row) initialisation ${\mathscr {T}}_0$; (second row) generated tree discriminating vessels given as initial conditions (blue), generated at the stage ${\mathscr {S}}_1$ (light blue) and stage ${\mathscr {S}}_2$ (red); (third row) variation of vessel radii along the generated network. Images generated with ParaView version 5.4 available at https://www.paraview.org/.

**Figure 7:**
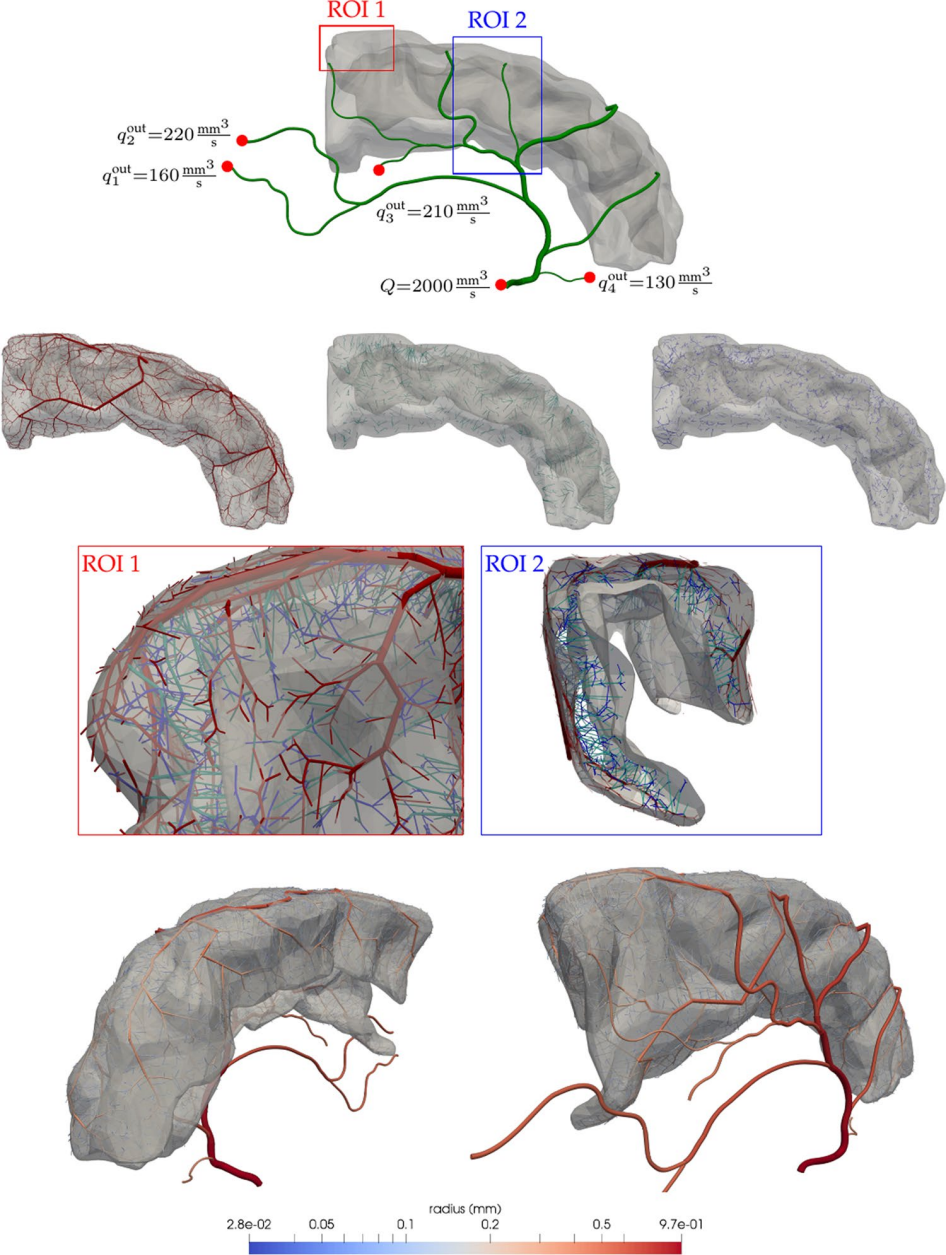
Grey matter vasculature in the superior frontal gyrus generated by a four-staged growth process: (first row) initial tree ${\mathscr {T}}_0$, illustrating the inflow and outflow constraints; (second row) from left to right, vessels corresponding to the pial network ($\mathscr {T}_1 \cup {\mathscr {T}}_2$), penetrating arterioles (${\mathscr {T}}_3$) and deep arterioles (${\mathscr {T}}_4$); (third row) superior-posterior part (left) and coronal slice at a middle section of the rostral-caudal axis (right), describing the architectural organisation of pial (red), penetrating (green) and deep (blue) vessels across the tissue; and (fourth row) final vascular tree depicting the vessel radius. Images generated with ParaView version 5.4 available at https://www.paraview.org/.

**Figure 8:**
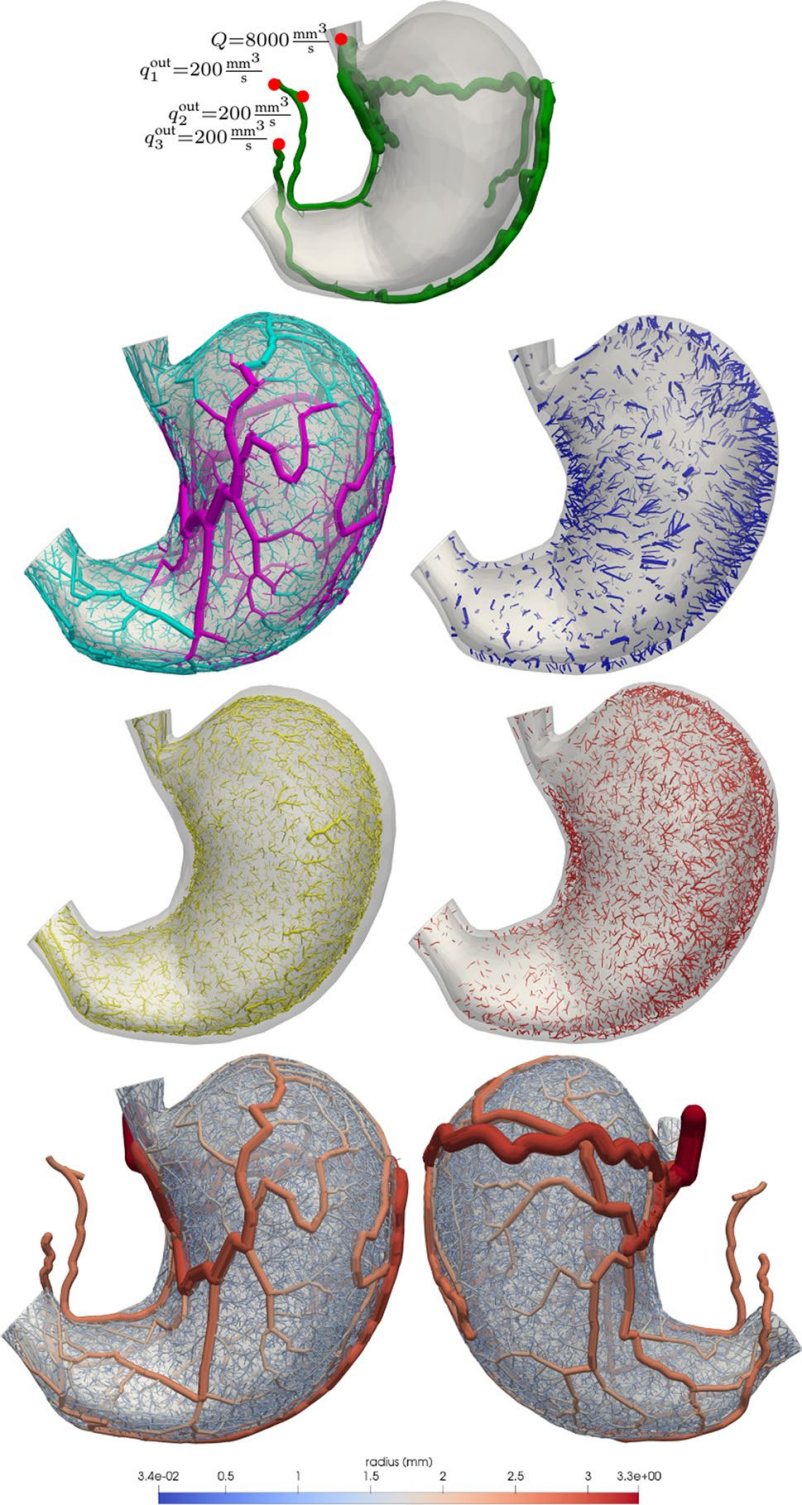
Stomach vasculature generated by a five-staged growth process: (first row) initial tree ${\mathscr {T}}_0$, including the inflow and outflow constraints; (second and third rows) from left to right and top to bottom, vessels corresponding to the serosa layer ($\mathscr {T}_1 \cup {\mathscr {T}}_2$), muscularis perforators (${\mathscr {T}}_3$), mucosa layer (${\mathscr {T}}_4$) and muscularis layer (${\mathscr {T}}_5$); (bottom row) final vascular tree depicting the vessel radius from anterior and posterior views of the stomach. Images generated with ParaView version 5.4 available at https://www.paraview.org/.

Parameters and stages of the DCCO method employed for each scenario described in this section are detailed in Table 1. The obtained vascular trees are illustrated in Figs. 6, 7, and 8, respectively.

It is important to point out that the parameters that define the series of growing stages are chosen to qualitatively mimic the dominant vascular architecture supplying the respective organs. This allows us to focus on the malleability of the framework to mirror complex anatomical arrangements of vessels.

### Inner retinal vascularisation

The inner retinal vascularisation, one of the vascular layers of the eye, is homogeneously vascularised with exception of the foveal region which is avascular. A physiologically accurate definition of its geometry was extracted from the OpenCMISS eye model (available at http://opencmiss.org/examples/a/eye/index.html), where we defined the position of the central retinal artery based on anatomical definitions of the ADAN model^74^ (see Fig. 6). This input vessel $v_0$ with radius $r_0=0.198\text { mm}$ and flow rate $Q=35\text { mm}^3{\mathrm{/s}}$ is consistent with experimental data^74^. To model the foveal avascular zone, an ellipsoidal region was defined at $3.5\text { mm}$ from the central retinal artery insertion towards the nasal direction occluding a circumferential region of $0.5\text { mm}$ of diameter.

In terms of vessel behaviour (as described in "Distribution, transport and perforator vessels" and "Vascular completion" sections), the input vessel was modelled as a *distal perforator* vessel and the generated vessels were *versatile distribution* vessels. The vascularisation process was developed in two stages with an initial condition ${\mathscr {T}}_0 = \{ v_0 \}$ and different symmetry ratio constraint parameters at each stage, which are defined as follows

19 $$\begin{aligned} \delta _1 = {\left\{ \begin{array}{ll} 0.4 &{} \text {for } l_{\text {bif}}<5,\\ 0.0 &{} \text {otherwise} \end{array}\right. }, \qquad \delta _2 = {\left\{ \begin{array}{ll} 0.8 &{} \text {for } l_{\text {bif}}<5,\\ 0.4 &{} \text {otherwise} \end{array}\right. } \end{aligned}$$

where $l_{\text {bif}}$ is the bifurcation level of $v_p$. Using this piece-wise symmetry constrain, we obtain a better coverage of the inner retinal surface by relaxing the symmetry ratio at the beginning of the process. At the second stage, in which higher symmetry ratios are enforced, we obtain more uniform arborisations. The remaining parameters used at each stage are detailed in Table 1.

Interestingly, the DCCO vascularisation generates four branches near the insertion point of the retinal artery creating a quadrant-based arrangement in the same manner that is observed *in vivo*^78^. In more distal sites, a uniform vascular pattern is delivered by the algorithm in agreement with anatomical descriptions^79^. This simple example shows the ability of the DCCO to reproduce the *in vivo* vascular patterns from a small set of constraints and stages.

### Brain cortex vascularisation

In this second case, we vascularise the grey matter tissue in the left frontal gyrus of a prototypical human brain. Its geometrical definition was based on a human brain reconstruction from BodyParts3D database (http://lifesciencedb.jp/bp3d - The Database Center for Life Science licensed under CC Attribution-Share Alike 2.1 Japan). As initial condition ${\mathscr {T}}_0$, an arterial tree rooted at the left anterior cerebral artery (l.ACA) was used. The anatomical description, flow constrains and relative location to the tissue are presented in Fig. 7 in green colour.

From the nine terminals of the initial tree ${\mathscr {T}}_0$, five perfuse the grey matter while four divert blood flow to other cerebral territories. To properly prescribe the outflow of the latter terminals, we use the strategy introduced in "Blood flow distribution" section constraining flows $q^{\text {out}}_i$ as described in Fig. 7 (see red-filled circles). The root of the l.ACA is defined with a radius of $r_0=0.965\text { mm}$ and flow rate of $Q=2000\text { mm}^3{\mathrm{/s}}$ consistent with data collected from experimental observations^74^. All vessels from the ${\mathscr {T}}_0$ were modelled as *non-branching* vessels with exception of the five terminals responsible for the perfusion of the grey matter, which were modelled as *distal perforator* vessels. Finally, the generated vessels were *versatile distribution* or *perforator* vessels (see Table 1).

The vascularisation process was modelled using four stages, the first two stages are responsible for the generation of pial vessels over the cortical surface, the third stage generates the penetrating arterioles and, lastly, the fourth stage generates deep arterioles. The first two stages were executed within a thin shell domain (the pial surface) $\Omega _{\text {pial}}$ generated as an outward extrusion of $\approx 1\text { mm}$ from the cortical surface. The following penetrating arterioles were grown as vessels branching off perpendicularly from trees ${\mathscr {T}}_1$ and ${\mathscr {T}}_2$. This was achieved by constraining the bifurcation angle within the range $[\theta _{\text {min}} = 80, \theta _{\text {max}} = 100]$. Finally, deep arterioles in the fourth stage were grown by branching from penetrating vessels. Stages ${\mathscr {S}}_1$ and ${\mathscr {S}}_4$ used the sprouting criterion to obtain a balanced growth of the tree, avoiding one branch taking over the perfusion process (as would be the case when using the volumetric functional). All parameters used at each of the mentioned stages are reported in Table 1.

**Figure 9:**
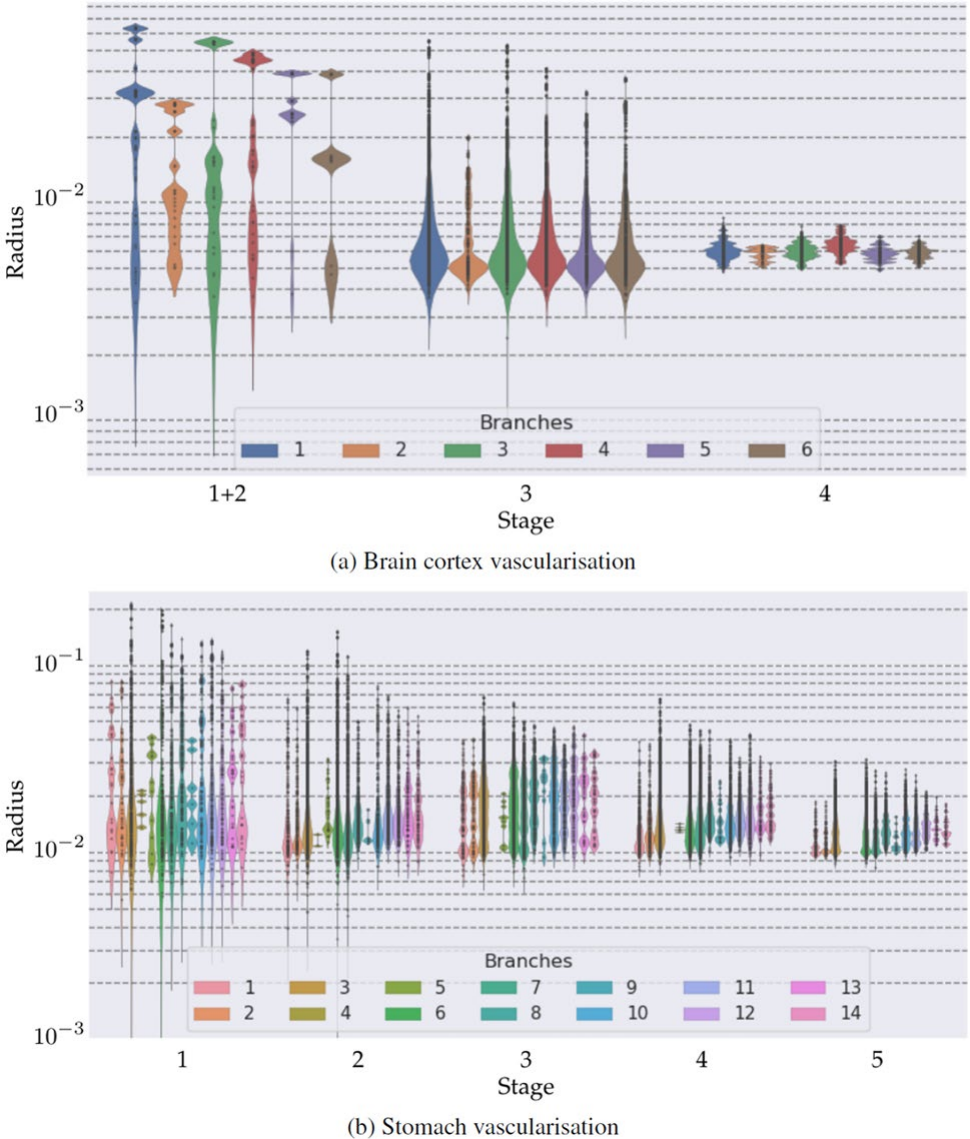
Radius distribution of vessels generated at each stage. Each colour corresponds to a different vessel branched form the initial tree ${\mathscr {T}}_0$. Black dots in the violin indicate a sample value for the radius. First stages present a multimodal distribution in which larger radius modes correspond to vessels transporting blood to neighbour regions vascularised at another stage and smaller radius modes correspond to terminal and distribution vessels. Plots generated with Seaborn library version 0.11 available at https://seaborn.pydata.org/.

The generated vascular tree reproduces several architectural features observed in experimental studies^80–83^. All main branches perfusing the gyrus are well developed providing significant blood flow to the tissue. In Fig. 9, the distribution of vessel radii is displayed for each one of the branches that are present in the initial cerebral tree from which the first stage of the DCCO algorithm is executed. Note that there is an additional branch in the central artery (centred in ROI2), which is caused by the *distal vessel* branching behaviour (see "Distribution, transport and perforator vessels" section) at the terminals of ${\mathscr {T}}_0$. Qualitatively similar distributions are observed for the different branches, with some quantitative differences resulting from the size of the corresponding vascular territories that each of them supplies. Also, the cortical vessels presented a homogeneous coverage of the gyrus surface, where its major pial vessels branch into penetrating vessels, which run inwards, nearly orthogonal to the gyrus surface. This results in the first generation of vessels which reach the grey matter. In turn, these penetrating vessels give rise to local arborisations of deep vessels. The final vascular tree homogeneously perfuses grey matter volume as is evidenced by the dispersion of the vessels generated in ${\mathscr {S}}_3$ and ${\mathscr {S}}_4$.

### Stomach vascularisation

As a final case, we built a vascularisation for the stomach following the anatomical description presented in Geboes et al. and Gorczyca et al.^84,85^. A geometrical description of the domain was obtained from BodyParts3D database (http://lifesciencedb.jp/bp3d). The three functionally different layers, namely serosa, muscularis and mucosa layers, were modelled using a thickness ratio of 1:4:3. As initial condition ${\mathscr {T}}_0$, we constructed a vascular tree rooted at the celiac artery (see Fig. 8, first row), eliminating the anastomoses between common hepatic and right gastric arteries and between right gastro-omental and gastric arteries. To avoid the degeneration of the right gastric and gastro-omental arteries, we prescribed distal outflow in these arteries as shown in Fig. 8. All vessels from ${\mathscr {T}}_0$ were modelled as *non-branching* vessels with exception of the perforator terminals located at the gastric, gastro-omental and splenic arteries which were modelled as *distal perforator* vessels.

The vascularisation process was modelled through five stages. The first stage is responsible for growing the main vessels of the subserosal plexus, which is achieved by optimising a sprouting cost functional. The strategy ensures a homogeneous proliferation of all perforator vessels from the posterior side, lesser, and greater curvatures alike. Then, a second stage completes the plexus using a volumetric cost functional, providing a more volumetric efficient arborisation. The third stage generates muscularis perforators from the subserosal plexus to the mucosa layer. These are straight vessels that run orthogonally to the layer, and which are generated by imposing angle constraints. The fourth stage relaxes the angle constraints and develops the submucosa plexus from the muscularis perforators using the conventional volumetric cost functional. Finally, the fifth stage vascularises the muscularis layer from both the muscularis perforators and the submucosa plexus.

The resulting vascular tree (see Fig. 8) is in qualitative agreement with the vascular architecture reported in Geboes et al.^84^ where the subserosal plexus delivers a homogeneous perfusion to the serosal layer, while perforators arising from this subserosal network feed the submucosa plexus. Lastly, the blood to the muscularis layer is provided simultaneously by such serosal perforators and by the submucosa plexus. The complex architecture of this organ rendered a more complex distribution of vessel radii at all stages due to the presence of vessels transporting blood to the neighbour at all regions (subserosal plexus vascularise submucosa, muscularis vascularise submucosa, submucosa vascularise muscularis). Figure 9 displays the distribution of vessel radii for each one of the already existing branches in the initial vascular network from which the rest of the tree is constructed; and this is shown for the five growth stages.

Vessels with such a transport role present larger diameters proportional to their effective distal resistance, producing a larger radius mode in the distributions than terminal vessels in the same region (depicted in Fig. 9). Finally, note that it was possible to emulate this complex topological configuration thanks to the concatenation of a few basic growing steps, within the framework of the proposed methodology.

**Table 1:** DCCO parameter setup for each of the vascularisation processes.

Stage	Domain	${\mathscr {P}}_{\text {geo}}$ $(\gamma ,\delta )$	${\mathscr {P}}_{\text {opt}}$ $(\nu , f_r, f_n, \Delta v)$	Initial condition	$N_{\text {T}_{\text {final}}}$	Cost functional	Vessel type	Angle constraint $\theta _{\text {min}},\phi _{\text {min}}$
**Inner retinal vascularisation**								
${\mathscr {S}}_1$	$\Omega$	$(3, \delta _1)$	(1.0, 0.9, 1, 7)	${\mathscr {T}}_0$	30	${\mathscr {F}}_{\text {vol}}$	Distribution	$20^{\circ },0^{\circ }$
${\mathscr {S}}_2$	$\Omega$	$(3, \delta _2)$	(1.0, 0.9, 1, 7)	${\mathscr {T}}_1$	5000	${\mathscr {F}}_{\text {vol}}$	Distribution	$20^{\circ },0^{\circ }$
**Brain cortex vascularisation**								
${\mathscr {S}}_1$	$\Omega _{\text {pial}}$	$(3, \delta _1)$	(0.01, 0.9, 100, 7)	${\mathscr {T}}_0$	20	${\mathscr {F}}_{\text {sprout}}$	Distribution	$20^{\circ },0^{\circ }$
${\mathscr {S}}_2$	$\Omega _{\text {pial}}$	$(3, \delta _1)$	(0.01, 0.9, 100, 7)	${\mathscr {T}}_1$	2500	${\mathscr {F}}_{\text {vol}}$	Distribution	$20^{\circ },0^{\circ }$
${\mathscr {S}}_3$	$\Omega$	$(3, \delta _1)$	(5, 0.9, 1, 7)	${\mathscr {T}}_1 \cup {\mathscr {T}}_2$	800	${\mathscr {F}}_{\text {vol}}$	Perforator	$20^{\circ },80^{\circ }$
${\mathscr {S}}_4$	$\Omega$	$(3, \delta _1)$	(0.001, 0.9, 200, 7)	${\mathscr {T}}_3$	4000	${\mathscr {F}}_{\text {sprout}}$	Distribution	$20^{\circ },0^{\circ }$
**Stomach vascularisation**								
${\mathscr {S}}_1$	$\Omega _{\text {serosa}}$	$(3, \delta _1)$	(0.01, 0.9, 100, 7)	${\mathscr {T}}_0$	500	${\mathscr {F}}_{\text {sprout}}$	Distribution	$10^{\circ },0^{\circ }$
${\mathscr {S}}_2$	$\Omega _{\text {serosa}}$	$(3, \delta _1)$	(0.1, 0.9, 100, 7)	${\mathscr {T}}_0 \cup {\mathscr {T}}_1$	4500	${\mathscr {F}}_{\text {vol}}$	Distribution	$20^{\circ },0^{\circ }$
${\mathscr {S}}_3$	$\Omega _{\text {mucosa}}$	$(3, \delta _1)$	(1, 0.9, 1, 7)	${\mathscr {T}}_1 \cup {\mathscr {T}}_2$	3000	${\mathscr {F}}_{\text {vol}}$	Perforator	$20^{\circ },0^{\circ }$
${\mathscr {S}}_4$	$\Omega _{\text {mucosa}}$	$(3, \delta _1)$	(1, 0.9, 4, 7)	${\mathscr {T}}_3$	10,000	${\mathscr {F}}_{\text {vol}}$	Perforator	$20^{\circ },0^{\circ }$
${\mathscr {S}}_5$	$\Omega _{\text {muscularis}}$	$(3, \delta _1)$	(1, 0.9, 1, 7)	${\mathscr {T}}_3 \cup {\mathscr {T}}_4$	5000	${\mathscr {F}}_{\text {vol}}$	Perforator	$20^{\circ },0^{\circ }$

The ${\mathscr {F}}_{\text {sprout}}$ was instantiated with parameters $c_v=1.0 \times 10^4, c_p= 0.5, c_d=1.0$ for the brain cortex and $c_v=1.0 \times 10^2, c_p= 0.5, c_d=2.0$ for the stomach case. Symmetry ratio constraint parameters $\delta _1$ and $\delta _2$ are presented in (19); and $\theta _{\text {max}}$ and $\phi _{\text {max}}$ are the supplementary angles of $\theta _{\text {min}}$ and $\phi _{\text {min}}$, respectively.

## Discussion

### Standard CCO and DCCO towards clinical applications

Standard CCO methods present shortcomings for incorporating anatomical knowledge of the vascular architecture and, also, to extend complex vascular trees taken from medical images. Versatility to encode vascular patterns is a desired feature to apply these algorithms in realistic scenarios encountered in basic and applied medical research and, ultimately, in the clinic.

**Figure 10:**
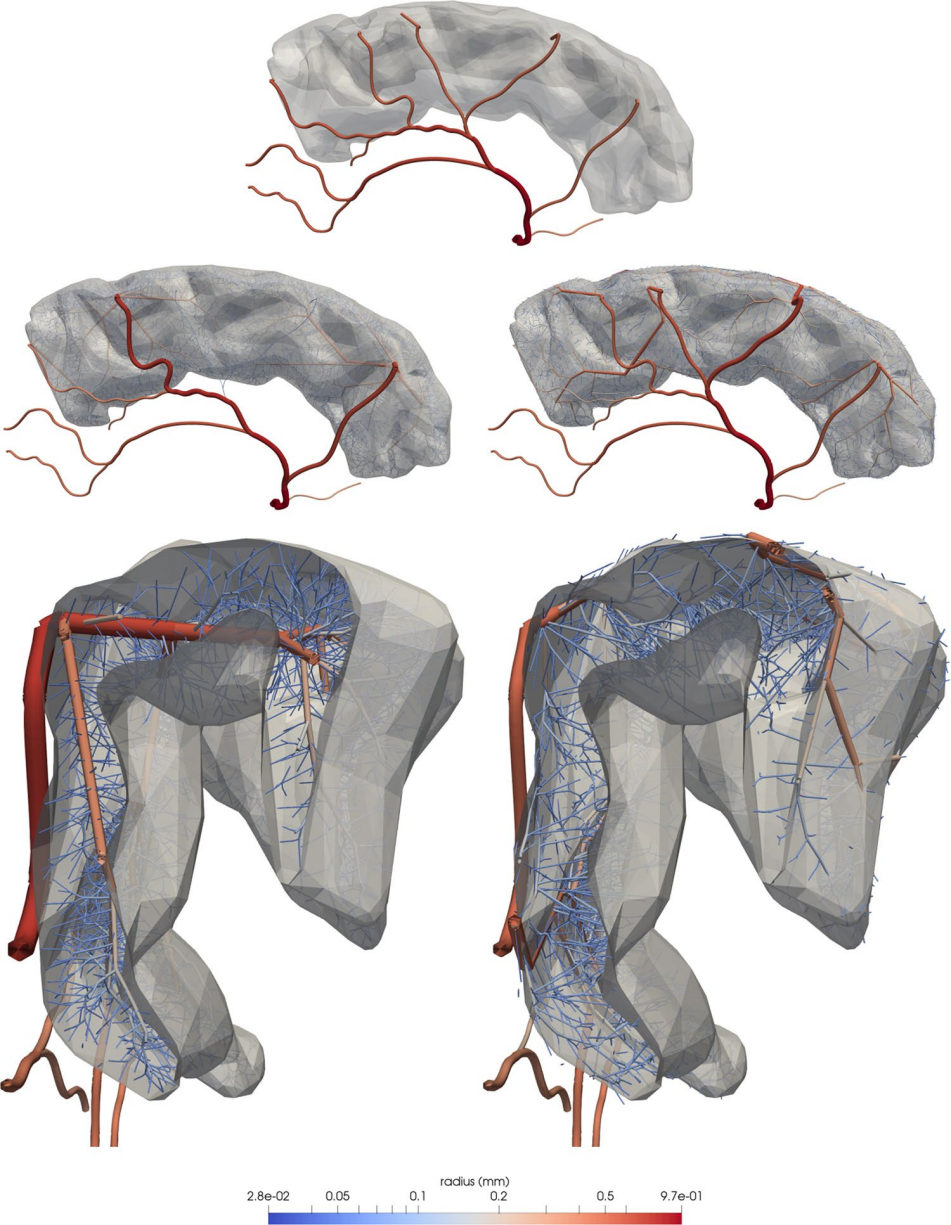
Comparison between standard CCO (left column) and the proposed DCCO (right column) methods. (Top) Initial vasculature; (middle) generated trees of 8000 terminal segments; (bottom) coronal slices at a middle section of the rostral-caudal axis. Images generated with ParaView version 5.4 available at https://www.paraview.org/.

To exemplify some of the standard CCO issues, we vascularised the left frontal gyrus from "Brain cortex vascularisation" section using a standard CCO algorithm and the proposed DCCO. As illustrated in Fig. 10, the CCO implementation presents subcortical-to-cortical perfusion, opposite to the realistic architecture, which can be reproduced by DCCO. Also, two of the five arteries given as the initial vasculature for standard CCO are not perfusing the tissue significantly, differently from the proposed DCCO. These two particular issues restrict usage of CCO for tissue- and patient-specific scenarios, respectively, hindering its application and validation in realistic simulations. In these aspects, DCCO offers a richer toolbox to model vascular architecture from scratch as well as to expand existing vascular networks in complex domains from a set of given vessels.

Noteworthy the versatility of DCCO has its impact in computational times as more constraints need to be checked when estimating the optimal bifurcation point in comparison with CCO (see line 17 and 20 from Algorithm 2 and 3 respectively). The particular cases presented in Fig. 10 were generated in 59.96 and 22.99 hours (wall clock time) for DCCO and CCO respectively, using an Intel Xeon Gold 5218 CPU at 2.30GHz with 128 GB RAM. It is important to mention that neither of the algorithms was optimised in terms of performance, and more efficient methods have been proposed in the literature^42^. Nonetheless, the main focus of our contribution was in the improvement of modelling capabilities of CCO method to deal with complex realistic scenarios and efficiency was out of the focus of this work. Still, the parallel generation of vast networks can be done by divide-and-conquer strategies trading off the optimisation of the cost function because of the decoupling of the divided sub-domains. Discussion of this approach will be addressed in future works.

### DCCO improvements for vasculature modelling

The techniques introduced in this work constitute a set of modelling tools to generate vascular networks compliant with anatomical knowledge, broadening the range of applicability and capabilities from models reported in previous works. Most traditional *in vivo* imaging techniques are only capable of rendering vascular structures with a pixel resolution of $\sim 0.5\text { mm}$^86^, hindering *in vivo* examination of microvasculature in healthy and pathological scenarios. In turn, *ex vivo* examination^30,81–84,87,88^ yields a detailed description of the microvascular architecture. As shown in the previous section, the proposed approach allowed the construction of different vascular architectures whose features resemble those reported *ex vivo*. We accomplished this by using a reduced number of growth stages with a proper rationale to select parameters and model each vascular stage. Moreover, the initialisation of the DCCO processes was performed with arterial geometries and blood flow measurements in the same manner as could be performed in an *in vivo* setup, initiating the process from medical images. As a result, the proposed methodology enables the generation of *in silico* models encoding refined architectural knowledge of the vascular tree delivering microvasculature models which are anatomically compatible with the available data. Moreover, if additional clinical data is available, further specialisation of the model can be achieved by using the blood flow distribution techniques presented in "Blood flow distribution" section to construct functionally compatible models.

As demonstrated from the examples reported in the previous sections, different tissues and organs feature specific vascular architectures associated with corresponding embryonic, developmental, and remodelling driving-forces. As a consequence, this imprints intrinsic characteristics onto the hemodynamic response and perfusion. Thus, it is expected that physiologically compatible models for representing the peripheral beds should reproduce the specific architectural patterns that account for the proper and specific functional response of the tissue. Hence we developed our approach to offer expressive modelling capabilities which enable the instantiation of a myriad of vascular layouts in the loci of interest, enhancing the customisation characteristics of previous CCO approaches, and aiming to improve the description of anatomical structures.

In such regard, the use of staged growth allowed us to model particular features at each step, simplifying the modelling of a complex architecture in a divide and conquer manner. At each stage, the goal was to focus on a particular anatomical feature such as balanced vascularisation from the inputs (sprouting cost functional), homogeneous vascularisation (volumetric functional), generation of perforator vessels across subdomains (perforator vessels), communication between domains (transport vessels), or constraining the geometrical characteristics at a given region or spatial scale (staged growth with different geometrical constraints). Also, the definition and interaction with initial conditions, such as patient-specific vascular networks reconstructed from MRI or CT imaging modalities, have been enhanced by: (i) generalising the tree representation as an *N*-ary tree; (ii) prescribing the bifurcation behaviour at the given (and generated) vessels (non-branching, distal, fixed, and versatile vessels); and (iii) prescribing outflows (*i.e.* known data) to adjacent vascular territories.

The analysis of the vessel radius distributions for the cases of the cortex and the stomach (see Fig. 9) show that each branch (present in the initial network from which the rest of the network is grown) features a specific distribution which is related to the geometrical region to be supplied, which is, in fact, unknown *a priori*. Moreover, the change in the network statistics experienced by each branch subtree through the growth stages is not a simple scaling, but is the result of the interplay between cost function, the topology of the domain to be perfused, and the set of parameters. This emphasises the fact that the proposed algorithm is endowed with the versatility and flexibility to embody networks of vessels with different probabilistic features concerning vascular geometry and connectivity.

### Opportunities and challenges

Nonetheless, there are still difficulties that must be addressed to reproduce certain anatomical scenarios. Vascularisation over curved shell-like domains such as organ surfaces or interfaces present a challenging task when using linear segments because they may fail to be constrained to the geometry of the vascular territory. The projection of these vessels over the domain would offer a solution although more expensive computations are required. In this work, we tackled this problem in the brain cortex case by extruding its surface inwards, generating a thin shell volume. Such a workaround yielded a proper cortical network to the detriment of a more computationally intensive optimisation procedure due to the large number of trials required to generate vessels inside the thin domain. Another challenge yet to be addressed is the generation of a vascular network with a graph-like topology. Anastomoses between arborisations are widespread throughout the different vascular scales in the cardiovascular system. The problem of introducing such capabilities to the DCCO (actually to any CCO-based approach) is that the heuristic optimisation strategy employed is not appropriate for graph structures. Furthermore, the way to proceed with the recruitment of anastomotic connections is not entirely clear.

It is important to mention that the methodology presented in this work aims at the generation of vascular networks from arteries/veins to pre-capillary levels, where the main features of the vascular network are reasonably described by a tree-like structure. The methodology has no theoretical impediments for the generation of large networks (*e.g.* up to $10^7$ vessels). However, a more practical approach in such a scenario would be to exploit a multi-scale paradigm, in which the proposed approach is employed to generate the larger scale vessels that describe the main vascular patterns (*e.g.* order of $10^4$ vessels) and generate the remaining vessels with faster filling techniques applied locally in predefined representative vascular domains^23,42^ to obtain a larger speedup. Note that the latter small-scale vessels are expected to be more homogeneous without different architectural traits, and the vascularisation could be executed in an “embarrassingly parallel” manner.

### DCCO for the modelling of realistic vascular trees

The vascularisation cases introduced in the previous section were chosen because they combine a series of difficulties such as the thinness and curvature of the domains, as well as the complex vascular architecture, which pose a challenge for the generation of an anatomically-realistic vascular network. We demonstrated that the proposed techniques cope with such difficulties by delineating simple, meaningful and rational growing stages, without the need for exhaustive tuneup of parameters. In fact, less intuitive geometrical parameters (related with symmetry ratio and Murray’s law coefficient) were fixed for all cases, to demonstrate that no fine-tuning of the parameters is required to model the construction of the vascular architecture. At this point, it is important to highlight that the main focus of this work was to present a new set of modelling tools with potential to generate complex models.

Finally, the generation of vascular networks in an automatic manner, either from scratch or from initial available data is of the utmost relevance to set the geometrical substrate for physiological models to be applied and explored. The anatomically-consistent disposition of the different generations and layers of arterial/venous vessels is crucial to embody physiological knowledge acquired at the different spatial scales in the human body, and to enable the study of mechanistic interactions among cellular function, microvascular physiology, tissue perfusion, and, eventually, whole-organ function.

## Conclusion

We introduced the aDaptive CCO (DCCO) as an approach for the generation of vascular networks with a set of functional and architectural traits. This work describes the algorithms that materialise DCCO and, also the rationale for modelling the necessary stages to achieve different and diverse anatomically-realistic representations of the vasculature.

Three complex anatomical scenarios were modelled –the retinal layer, a section of the superior frontal cortex, and a three-layered description of the stomach– reproducing experimental observations reported in previous works. To the best of our knowledge, the anatomical detail achieved with the proposed approach is not attainable with other CCO-based techniques.

In addition, further extensions were made to integrate the automatic vascularisation process with available data, such as that obtained from medical images or detailed models of the cardiovascular system. These features allow us to use DCCO in patient-specific geometries, enabling new research opportunities to explore the role of small-scale vessels and their physiological characteristics into different clinical scenarios.
